# Unlocking the Full Potential of Jumping Condensation on Microstructured Surfaces

**DOI:** 10.1002/advs.78273

**Published:** 2026-10-14

**Authors:** Mariia S. Kiseleva, Tytti Kärki, Mika Latikka, Parham Koochak, Sakari Lepikko, Maja Vuckovac, Tomi Koskinen, Ramesh Raju, Ville P. Jokinen, Jiazheng Liu, Nenad Miljkovic, Jaakko V. I. Timonen, Ilkka Tittonen, Robin H. A. Ras

**Affiliations:** ^1^ Department of Applied Physics Aalto University Espoo 02150 Finland; ^2^ Centre of Excellence in Life‐Inspired Hybrid Materials (LIBER) Aalto University Espoo 02150 Finland; ^3^ Department of Electronics and Nanoengineering Aalto University Espoo 02150 Finland; ^4^ Department of Chemistry and Materials Science Aalto University Espoo 02150 Finland; ^5^ Department of Mechanical Science and Engineering The Grainger College of Engineering University of Illinois Urbana‐Champaign Urbana Illinois USA; ^6^ Materials Research Laboratory University of Illinois Urbana‐Champaign Urbana Illinois USA; ^7^ Department of Electrical and Computer Engineering University of Illinois Urbana–Champaign Urbana Illinois USA; ^8^ International Institute For Carbon Neutral Energy Research (WPI‐I2CNER) Kyushu University Fukuoka Japan; ^9^ Air Conditioning and Refrigeration Center Department of Mechanical Science and Engineering University of Illinois Urbana‐Champaign Urbana Illinois USA; ^10^ Institute for Sustainability, Energy, and Environment (iSEE) University of Illinois Urbana Illinois USA

**Keywords:** anti‐fogging, droplet, heat transfer, jumping condensation, microstructuring, superhydrophobicity, water harvesting

## Abstract

Water condensation on superhydrophobic surfaces can generate spontaneous droplet jumping, enabling rapid condensate removal and improved thermal and mass transfer. Although this effect has been extensively demonstrated on densely packed nanostructures, the capability of microscale textures to support jumping condensation remains poorly understood. By systematically varying cone pitch within a series of microcone arrays with comparable cone geometries, we observe previously unreported spacing‐dependent transition between jumping and non‐jumping regimes. In the jumping regime, we identify a geometric threshold below which sub‐10 µm droplets are rapidly removed, and above which jumping is suppressed, resulting in slower dynamics and larger departing droplets. In situ optical and environmental scanning electron microscopies provide evidence supporting a mechanistic interpretation of this transition: dense arrays favor full Cassie droplets, which depart cleanly, while wider spacing favors partial Cassie droplets that retain a localized wet region initiating new nucleation. Our findings establish a mechanistic link between the morphology of condensing microdroplets and the macroscopic jumping dynamics that govern performance in anti‐fogging, water harvesting, and heat‐transfer applications.

## Graphical abstract

Microcone arrays exhibit a spacing‐dependent transition between jumping and non‐jumping condensation. Dense microstructures favor full Cassie droplets, which depart cleanly, while wider spacing favors partial Cassie droplets that retain a localized wet region initiating new nucleation. These findings establish a link between microdroplet morphology and macroscopic condensation dynamics, providing insights for future microstructured surface design.

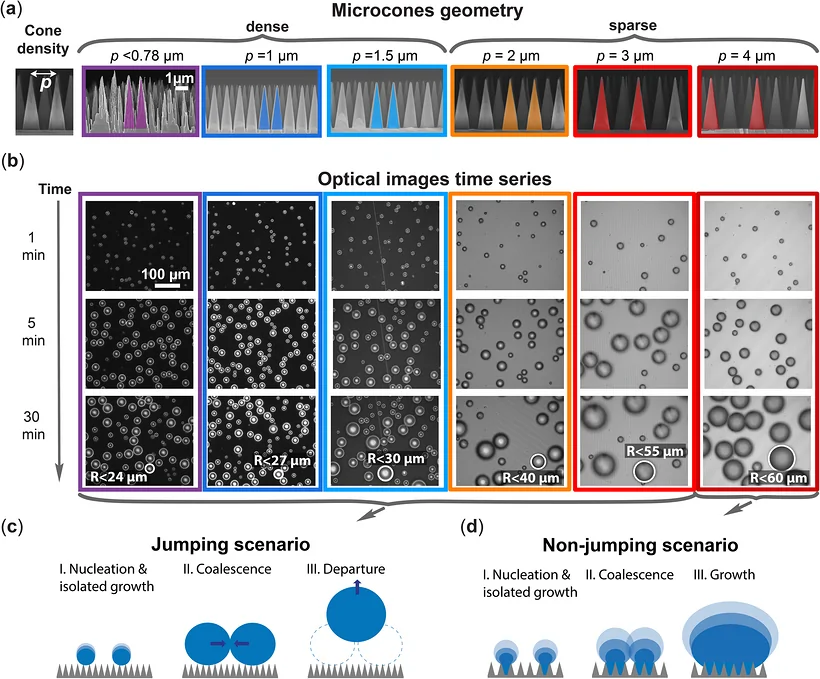

## 1 Introduction

Coalescence‐induced droplet jumping on superhydrophobic surfaces provides an efficient mechanism for condensate removal. When neighboring droplets merge, excess surface energy propels the droplet away [1], initiating repeated cycles of nucleation, droplet growth, coalescence, and departure. This process minimizes surface coverage, making it attractive for anti‐dew [2, 3], anti‐icing [4], anti‐biofouling [5], and anti‐particulate fouling [6, 7] applications. Continuous renewal of nucleation sites also enhances heat transfer [8, 9, 10] and accelerates water collection [11]. Despite these advantages, practical implementation is limited. Droplet dynamics are challenging to control, and eventual flooding of surface cavities leads to pinned Wenzel droplets and diminished jumping efficiency [9, 12, 13].

Jumping condensation has been studied extensively on stochastic nanotextures [9, 13, 14]. Despite scalable fabrication methods, these surfaces offer limited control over feature geometry and, consequently, condensation performance. Dense nanocone arrays inspired by cicada wings [5] were found to efficiently shed sub‐10 µm droplets [2, 3]. Unlike pillar arrays, droplets resting on the small tips of nanocones adopt a stable Cassie state, promoting active jumping across a wide range of cone's geometries (sizes, apex angles, and tip shapes). Micrometer‐scale structures enable more robust superhydrophobic surfaces fabricated using scalable methods (e.g., maskless etching [15], projection UV or nanoimprint lithography, and high‐resolution 3D printing [16]).

Translating the advantages of conical geometries to microscale structures, where droplet size is comparable to the texture scale, requires a mechanistic understanding of droplet morphology on microstructured surfaces, as it governs jumping dynamics and ultimately application performance. Despite numerous reports of jumping condensation on microstructures with nanoscale roughness [10, 17, 18, 19, 20], where droplets often adopt Cassie state at the nanoscale while remaining confined within the microstructure, studies isolating droplet morphology on microtextured surfaces without additional nanoscale roughness remain limited, mainly to pillar arrays [21, 22] and, more recently, dense conical arrays [23].

On microstructured surfaces, droplets can adopt suspended (full Cassie) or partially wetted (partial Cassie) states [22]. Both can jump, but they differ in growth rates, nucleation behavior, and heat‐transfer characteristics. Partial Cassie droplets grow faster and initiate new nucleation [24], influencing subsequent jumping dynamics and droplet size distribution.

Here, we combine well‐defined microconical arrays with in situ optical, confocal, and environmental electron microscopies to track microdroplet formation and evolution. Within the investigated sample series and environmental conditions, by systematically varying cone spacing, we reveal how microstructure density influences droplet morphology, coalescence‐induced jumping, and overall condensation performance. For the first time, we identify a sharp, spacing‐dependent transition between jumping and non‐jumping regimes on microscale conical arrays. In the jumping regime, sub‐10 µm droplets are efficiently removed below the critical spacing and suppressed above it. We translate these findings into a quantitative geometry–wetting design map that links microstructure spacing, droplet morphology, and nucleation density, offering a basis for future exploration of tailored surface design for anti‐fogging, water harvesting, and heat‐transfer enhancement. The use of microscale rather than nanoscale features may offer opportunities for improved mechanical robustness and implementation using scalable fabrication approaches; however, robustness and scalability were not directly evaluated in the present study.

## 2 Results and Discussion

### 2.1 Cones Geometry and Wetting Characterization

To investigate the effect of surface geometry on jumping dynamics and droplet morphology, we chose sharp microcones of similar size (cones’ height *h*  =  4.03 ± 0.35 µm; diameter *d*  =  1.23 ± 0.2 µm, opening angle *β * =  17.58 ± 3.17 °, tip diameter *d_t_*  =  0.11 ± 0.02 µm) systematically varying spacing *p* (peak‐to‐peak) between cones (Figure 1a and Table S1). We prepared a series of samples with regular, hexagonally packed cones (*p* = 1 – 4 µm), as well as irregular, densely packed array of sharp cones (so‐called “black silicon”, *p* < 0.78 µm). For all samples, we calculated solid fraction *φ* and surface roughness *R_f_* (Table S1). All microstructured samples (Note S1 on sample preparation) were coated with perfluorooctyltrichlorosilane (PFOTS) by chemical vapor deposition (CVD) method [25]. The coated planar surface exhibited advancing/receding contact angles of *θ_a_ /θ_r_ =* 117.9 ± 0.56 / 110 ± 0.3*°* with contact angle hysteresis *CAH* = 8 ± 1.6*°*. By combining microstructuring and chemical modification, all samples became superhydrophobic with contact angle higher than 160° (Figure S1a, and Note S2 on Contact angle measurement). Additionally, we used a scanning droplet adhesion microscope – SDAM (Note S3) [26] to measure the adhesion of droplets with a diameter of 1.4 mm (Figure S1b). Adhesion force scaled with the solid fraction, with smaller adhesion corresponding to sparser cones, consistent with previously reported friction measurements [27]. Macroscopic drops used for wetting characterization primarily interact with the tops of cones. Consequently, denser regular cone arrays lead to higher contact angle hysteresis and stronger adhesion forces, while “black silicon” with hierarchical nanostructures on microcones exhibits lower hysteresis and adhesion due to their complex multiscale surface topography.

**FIGURE 1:**
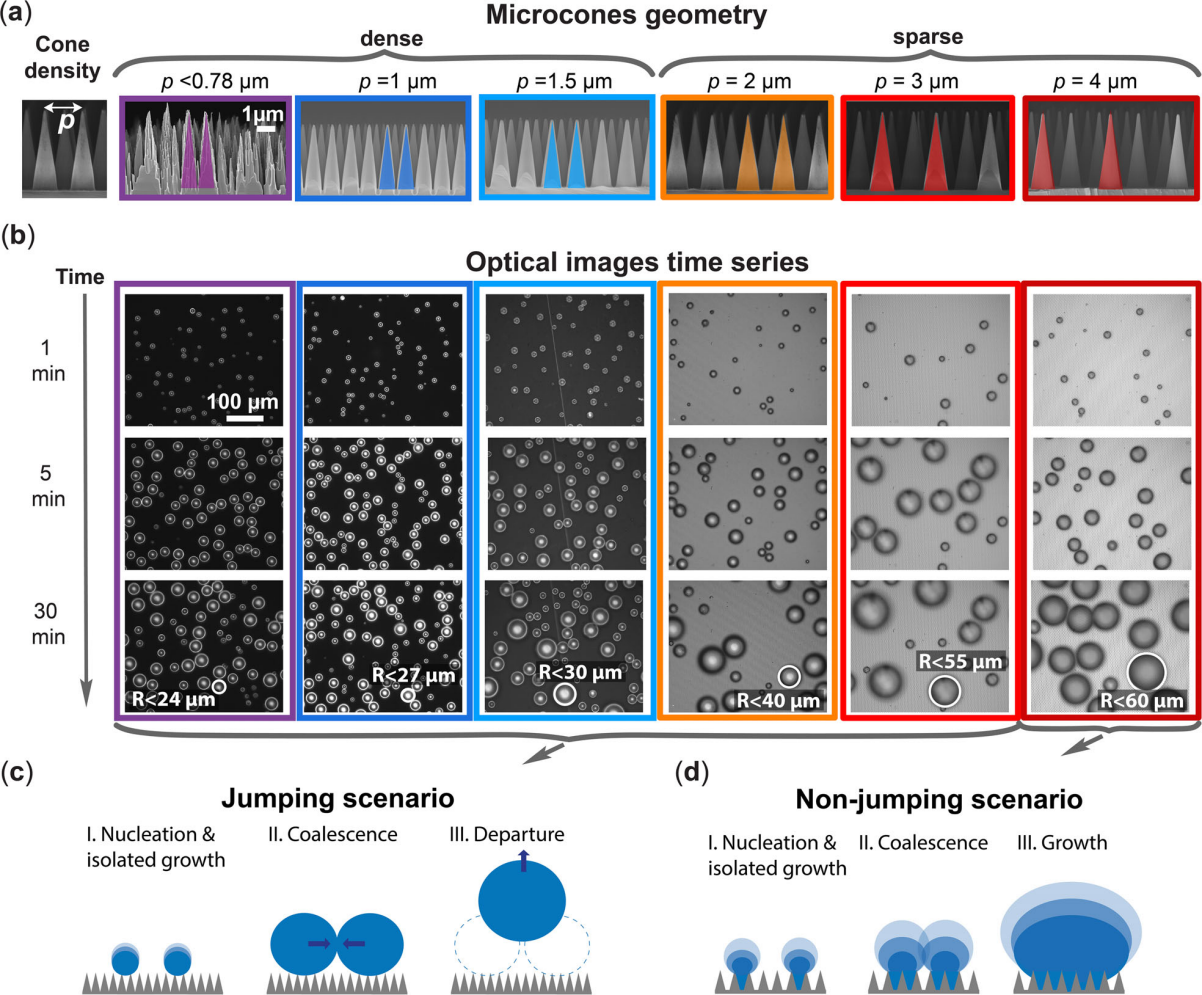
Evolution of condensation on microcones: visualization and main scenarios (a) SEM micrographs of dense irregular cones of "black silicon" with spacing *p* < 0.78 µm (violet) and hexagonally packed cones with spacing *p* = 1, 1.5, 2, 3, and 4 µm (blue, light blue, orange, red, dark red, respectively). Samples with *p* < 2 µm are referred to as dense, and those with *p* ≥ 2 µm as sparse. Scale bar of 1 µm applies to all panels. (b) Time series of breath figures at 1, 5, and 30 min of the condensation process on an array of microcones. Maximum droplet radii after 30 min of experiment are indicated with white circles. Scale bar 100 µm applies to all panels. Schematics of the dominant droplet coalescence scenario for (c) "black silicon" and cones with *p* = 01, 1.5, 2, 3 µm, which show droplet jumping, and for cones with (d) *p* = 4 µm, which show no jumping.

### 2.2 Condensation Performance and Jumping Dynamics

Condensation patterns on prepared microconical surfaces were visualized using an inverted portable microscope in controlled ambient temperature and humidity (Figure S3 and Note S4). Due to micrometric size of condensing droplets considerably smaller that capillary length, the gravity, i.e. sample orientation does not play a significant role in jumping dynamics [3]. To avoid influence of droplets landing back on or sliding from the upper part of the sample, we used an upside‐down sample orientation. In the time series of optical images, we observed the evolution of breath figures on surfaces with variable density of the cones (Figure 1b; Video S1). Time series were analyzed with a custom‐made image processing algorithm (Note S5) tracking each condensing droplet and defining its radius, position, circularity, etc. The post‐processing algorithm enabled identification of merging and jumping events during the experiment.

Samples with densely packed cones having spacing *p* < 2 µm (“black silicon”, hexagonally packed cones with *p* = 1 µm and *p* = 1.5 µm) will be referred to as dense cones, while samples with *p* ≥ 2 µm will be referred to as sparse cones (with *p* = 2 – 4 µm spacings). Dense cones appeared black due to high absorption of incident light. Conversely, sparse cones appear grey due to light reflection from flat areas between the cones, with individual cones being clearly visible on micrographs.

The typical condensation cycle includes three steps: (1) nucleation of multiple randomly distributed droplets, (2) growth of isolated droplets without coalescence, (3) coalescence of neighbouring droplets, which may result in either merging or jumping. In the jumping scenario (Figure 1c), after coalescence, two or more droplets gain sufficient kinetic energy to depart from the surface. Upon departure, the cycle repeats continuously. In the merging scenario (Figure 1d), the resulting droplet continues to grow until the next coalescence.

All samples except the sample with *p* = 4 µm exhibited the jumping condensation scenario. In the early stage of the condensation process, small droplets may merge due to strong pinning at the local defects, but resulting droplets will eventually be removed by the jumping mechanism. In contrast, on cones with *p* = 4 µm, condensing droplets were unable to jump, they merge and remain on the surface instead behaving similarly to condensing droplets on flat untextured surface.

Figure 2a shows that the nucleation density, *N_s_* (defined here as the droplet number per projected surface area), decreases with increasing spacing. Although nucleation occurs at the nanoscale and optically observed droplets form through coalesced nanodroplets, we use the term “nucleation density” (*N_s_*) for simplicity, instead of the more precise droplet number density (*DND*). Maximum (peak) values of nucleation density for all samples from dense to sparse, were 1543 ± 1122, 777 ± 305, 704 ± 341, 306 ± 239, 144 ± 83, 136 ± 23 mm^−2^ respectively. For comparison, the maximum *N_s_* for planar silicon functionalized with PFOTS using the same deposition protocol under similar condensation conditions was 350 ± 40 mm^−2^ [25]. Calculated nucleation density in relation to spacing between surface features *<L>/p*  [21] (*<L>* is mean separation distances between droplet centres defined by Poisson distribution $<L>=1/2\sqrt{N_{s}}$) revealed that *<L>/p* exceeded 10 across all our microconical samples indicating that nucleation did not occur within every structural cell.

**FIGURE 2:**
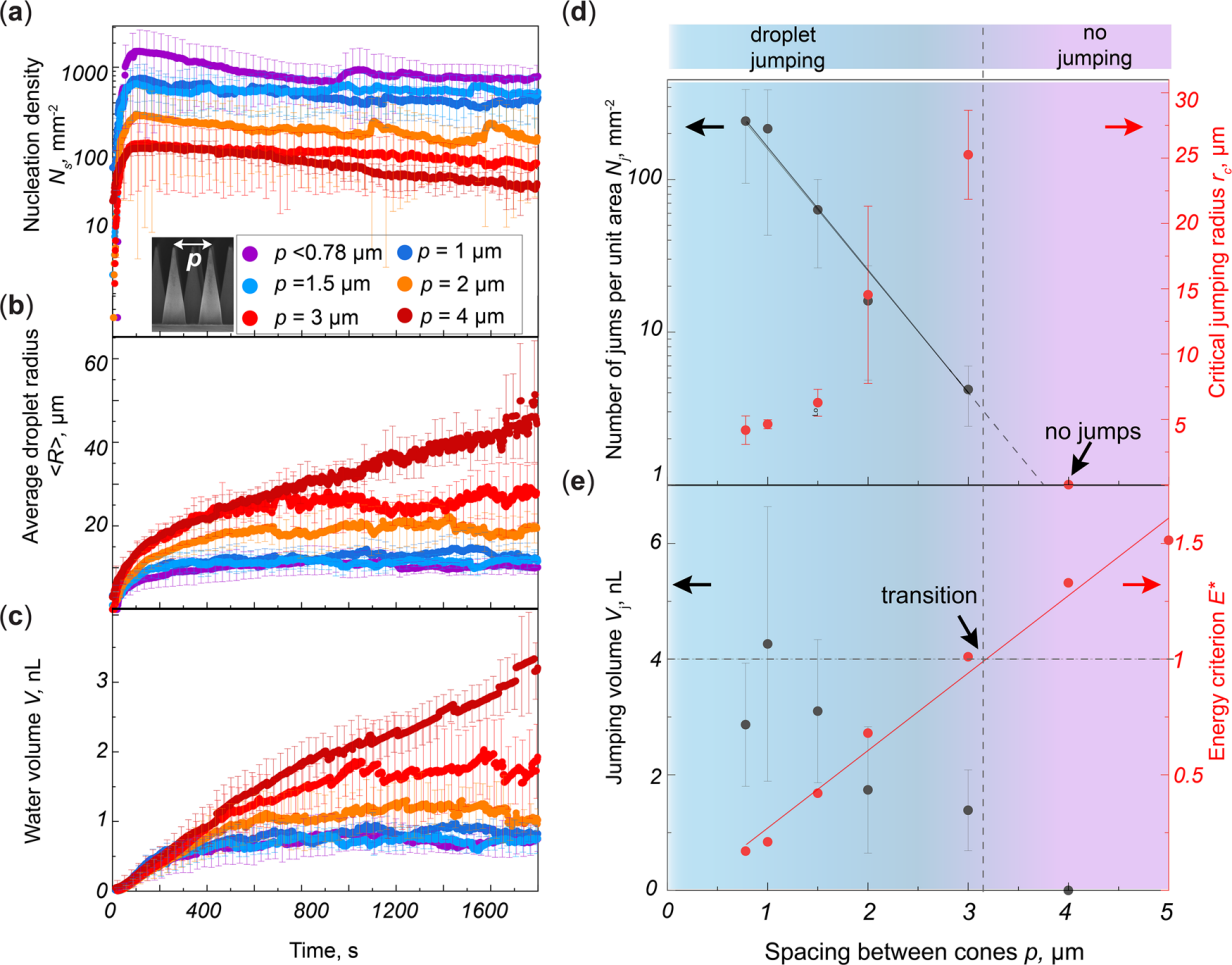
Droplet jumping efficiency on microcones: from active to inhibited jumping. Kinetics of (*a*) droplet number density in logarithmic scale, (b) average droplet radius *<R>*, (c) volume of remaining water on the sample. Insert in (a) depicts spacing *p* between cones; Same legend for panels (a–c). Error bars correspond to the standard deviation of at least 4 independent measurements (*n* = 4…5) on different samples at supersaturation level *S*  =  1.6 ± 0.2. (d) Number of droplet jumps per unit area in 30 min (log scale; left side) and critical jumping radius *r_c_* (right side); (e) jumping volume *V_j_* per unit area in 30 min (left side) and dimensionless energy criterion E^*^ (right side) as a function of spacing *p*. The total number of events was quantified over an experimental period of 30 min.

Variation in nucleation density with feature density is not sufficiently discussed in the literature due to large variation in surface design, mostly irregular nanostructured [13, 24], and inconsistent experimental conditions [28, 29]. We suggest that in microscale roughness cases, higher nucleation density on dense cones can be explained by the larger total surface area compared to sparse cones, and the increased feature density, which enhances the chances of nanoscopic droplets merging to form a stable droplet. In contrast, on sparse cones having a smaller total area, nanoscopic droplets are less likely to encounter other nanodroplets. An overall decrease in three‐dimensionally normalized nucleation density (Table S2) is observed with increasing cone spacing, despite some variation between individual types of samples, indicating the complex nature of droplet formation on microstructured surfaces and highlighting the importance of further investigation.

Active droplet removal on samples with jumping condensation keeps surface coverage *Φ* (proportion of apparent area covered by droplets to projected imaging area) below 35% with rare spikes up to ca. 40% for sparse cones (Figure S4a). The maximum droplet radius growth is limited by jumping condensation (Figure S4b). The droplet size distribution shifts toward larger radii as the spacing increases (Figure S5). As the result, the average droplet radius <*R*> is significantly smaller for dense cones compared to sparse ones, i.e. ca. 10 µm vs. 22 – 46.2 µm (Figure 2b), respectively. Since the volume of residing water on the sample *V* is proportional to the cube of the droplet radius (the droplets are mostly spherical, as will be described later), larger droplets contain greater volumes despite their lower distribution density. Consequently, *V* is significantly larger on sparse cones than on dense ones (Figure 2c).

The kinetic curves of <*R*> and *V* exhibit similar shapes, consisting of linearly increasing segments associated with droplet nucleation, growth, and coalescence, followed by a plateau caused by droplet removal through jumping (small spikes on the plateau curves are attributed to droplet removal events). Jumping condensation limits both the droplet size and total condensed volume. In contrast, for samples with *p* = 4 µm, where no jumping occurs, <*R*> and *V* increase linearly.

We extracted the frequency of droplet removal and the size of removed droplets by analyzing droplet jumping dynamics. We found that number of jumps *N_j_* (total number of jumps per unit area in 30 min) decreased exponentially (linearly in log scale) with increased spacing between cones, reaching zero for cones with *p* = 4 µm. The droplet jumping rate *J*, defined as the proportion of jumping events relative to all coalescence events, increases steeply for dense cones (Figure S6), indicating that droplets are removed at the very early stage of condensation. On the contrary, on sparse cones, droplets undergo a merging stage, delaying jumping. The critical jumping radius *r_c_* (radius of droplets at which the jumping rate reached 50%) (Figure 2d and Figure S7), increasing from ca. 5 µm for dense cones to 15 and 25 µm for sparse cones, while there is no jumping for the sample with *p* = 4 µm. The jumping volume *V_j_* over 30 min of experiment (Figure 2e), which can be considered as an integral parameter considering the number of jumps as well as the radii of jumping droplets, goes in line with the number of jumps trend: *V_j_* decreases linearly while spacing increases. Notably, for both dense cones and the 2 µm spacing sample, *V_j_* exceeds the volume of remaining condensed water on the sample (Figure S8), whereas for the *p* = 3 µm and *p* = 4 µm samples, the volume of remaining condensed water is greater.

Time‐resolved analysis of the jumping volume *V_j_* (*t*) and total volume *V_tot_* (*t*) (Figure S9a, b) showed that dense cones provide nearly instantaneous water removal that remains stable throughout the experiment due to the frequent jumping of small droplets, resulting in a jumping volume flux of 0.1 – 0.2 nL/min. In contrast, on sparse cones the onset of droplet jumping is delayed, and the jumping volume flux fluctuates strongly around 0.2 nL/min due to the relatively infrequent jumping of larger droplets. Dynamic ratio *V_j_(t)/V_tot_(t)* (Figure S9c) reveals that for dense cones volume of jumping droplets constantly fluctuates on average between 10% to 20% from the total volume of condensed water in a moment of time, while for sparse cones this ratio is notably smaller and fluctuates irregularly around 10%.

Trends for *N_j_* and *V_j_* align very well with predictions of energy criterion [21] (Figure 2e right side, Table S1) $E^{*}=cos\theta_{a}^{CB}\;/\;cos\theta_{a}^{W}=-1/\left(R_{f}cos\theta_{a}\right)$, where *cos*θ_*a*_ – cosine of the intrinsic advancing contact angle, and *R_f_* is the surface roughness. For E^*^ > 1, the Wenzel state is favorable, while E^*^  < 1 Cassie state prevails. A transition point occurs at E^*^  =  1, corresponding to a *p* = 3 µm spacing, where jumping still occurs, unlike for the next sample with *p* = 4 µm spacing. Predicted by simple theoretical parameter E^*^, the transition in droplet morphology can shed the light into changes of condensation performance with increasing the spacing and explain failure of jumping for the sample with *p* = 4 µm.

### 2.3 Studies of microdroplet morphology

To explain the difference in jumping performance between dense and sparse cones, we analyzed microdroplet growth using environmental scanning electron microscopy (ESEM) (Note S6). On dense cones, especially “black silicon”, very small droplets reached contact angles above ∼170°, enabling early‐stage removal (Figure 3a). In contrast, droplets on sparse cones required larger radii to exceed the ∼160° threshold for jumping [14]. ESEM further revealed two distinct morphologies: [22] Cassie droplets (Figure 3a, insert, Figure S10a–c), and partial Cassie droplets (Figure 3a, insert, Figure S10d). Partial Cassie droplets dominated on sparse cones, consistent with their reduced jumping efficiency.

**FIGURE 3:**
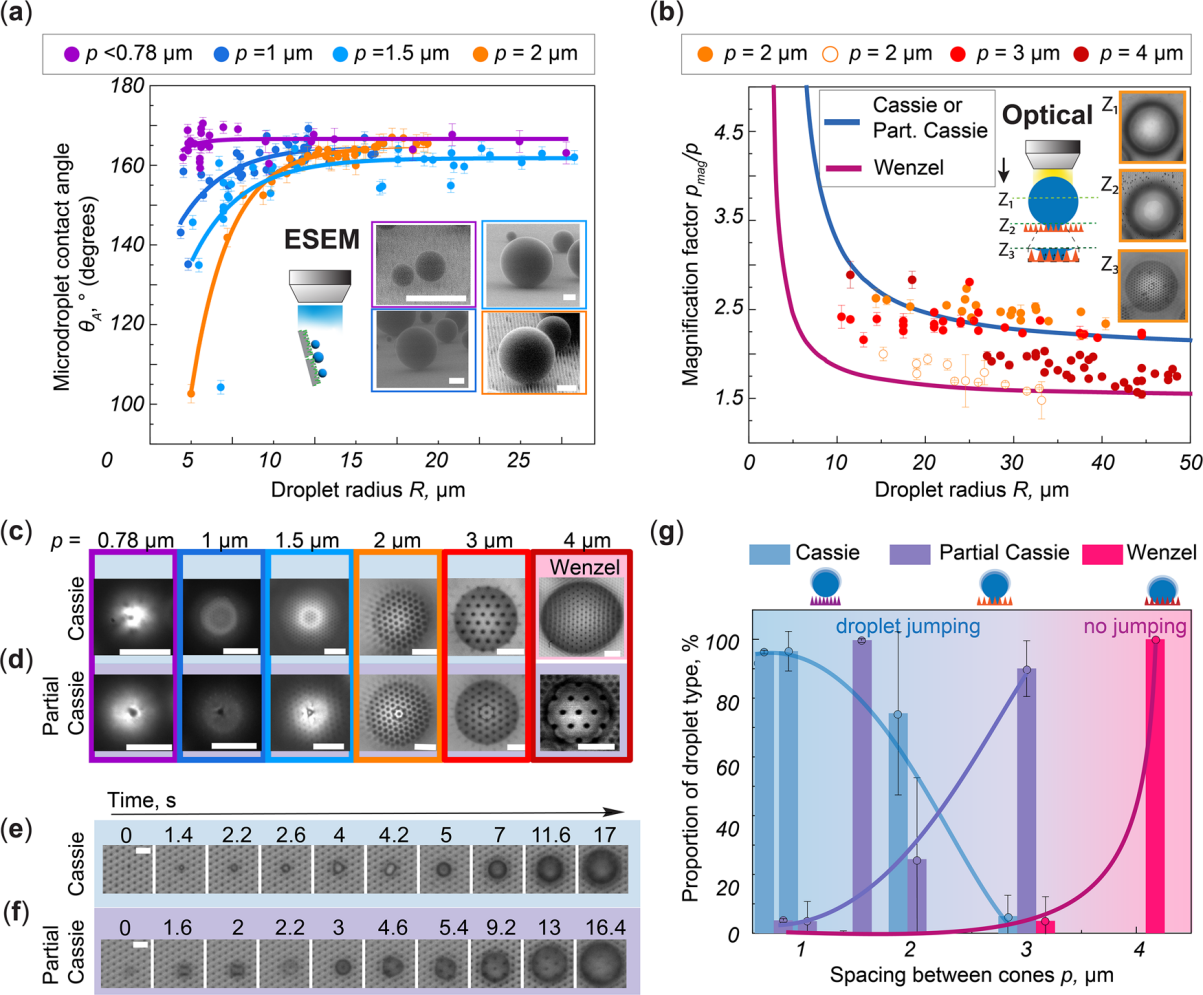
Transitions in microdroplet morphologies: from jumping Cassie to non‐jumping Wenzel droplets toward intermediate partial Cassie state. (a) Microdroplet visualization using environmental SEM: contact angle as function of droplet radius, standard deviation was defined from 10 measurements of the same droplet. The inserts are representative images of microdroplets on surfaces with variable spacing between the cones. Scale bar is 10 µm. (b) Magnifying effect of condensing droplets on sparse cones (*p* = 2, 3, 4 µm, hollow orange circles *p* = 2 µm transitioned to Wenzel case). Magnification factor is defined as *p*_mag_/*p*, where *p*_mag_ is the apparent distance between nearest cones measured through the droplet and *p* is the pitch on the dry surface. Samples with *p* = 2, 3 µm follow Cassie or partial Cassie magnification model (blue solid line), whereas sample with *p* = 4 µm follows Wenzel model (magenta solid line). Insets show schematic illustration of the optical setup used to image droplets at different focal planes (Z_1_, Z_2_, Z_3_) and perform magnification analysis. Optical microscopy visualization of microdroplets in (***c***) Cassie, (d) partial Cassie having fully wetted area surrounded by air layer, and Wenzel states on samples with variable spacing. Early stages of droplet growth on sparse cones with (e) Cassie and (f) partial Cassie morphologies. Scale bar is 4 µm. (g) Proportion of each droplet type after 30 min of experiment defined by changing focus, solid lines (blue, violet and pink) are guide for the eye presenting global trend across range of samples. Error bar is standard deviation calculated from at least 4 measurements (*n* = 4…20).

Along with ESEM we used non‐invasive optical imaging from the top, varying the focal plane of the microscope [30, 31], we were able to visualize the contact area of the droplet and the surface (Figure 3b, insertion). The advantage of ‘sparse’ arrangement is that light can be reflected from the flat area between bases of adjacent cones, whereas densely packed cones prevent such reflection. Optical microscopy of droplet morphology was validated by magnifying factor analysis considering optical models of Cassie and Wenzel droplets (Figures S11‐S13), where condensing droplets act as magnifying lenses (Discussion S1), and further confirmed using confocal laser scanning microscopy (Note S7). The analysis shows that both Wenzel and Cassie droplets magnify the underlying surface (Figure S11a,b). However, Cassie droplets refract light at both the top and bottom interfaces, causing the image to form deeper below the surface and thereby enhancing the magnification effect (Figure S11b). As a result, when observing textured surfaces through microscopic droplets, distinct differences in magnification between Wenzel and Cassie states become apparent (Figure S11c). To quantify this effect, we calculated the expected magnification for droplets of different radii (*R*) and wetting states and compared these predictions with experimental measurements (Figure 3b). Samples with *p* = 2, 3 µm follow Cassie or partial Cassie magnification model, whereas sample with *p* = 4 µm and non‐jumping due to defects sample with *p* = 2 µm follow Wenzel model. The obtained optical imaging data corroborated the ESEM measurements: we observed Cassie (Figure 3c), partial Cassie (Figure 3d), and Wenzel (Figure 3c) droplets.

When optically scanning a Cassie droplet (Figure 3c) by varying focal plane, the interface remains continuous as the droplet rests on cone tops with air beneath. In contrast, scans of a partial Cassie droplet (Figure 3d) show clear intensity variations revealing a circular or triangular wetted spot with defined boundaries, marking the transition from a water–air to a water–solid interface, observed at different focal planes for different droplet sizes. For dense cones, the stable configuration of wetted spot (Figure 3d) is a triangle between adjacent cones, while for sparse ones (*p* = 2, 3 µm) it is hexagon with one cone inside, and almost a circle of 16 µm in diameter for *p* = 4 µm spacing sample. Interestingly, on a non‐jumping sample with *p* = 4 µm condensing droplets are initially in a partial Cassie state that collapse into the Wenzel state after the first merging event.

For further validation of droplet state observation, we combined focal‐plane scanning (over an area of at least 0.62 mm^2^ free f defects and contamination after 30 min of condensation) with registering subsequent event after coalescence, namely jumping or merging. In the case of jumping droplets, we further distinguished between clean departure from the surface and departure followed by renucleation of a droplet at the same location. We constructed a diagram illustrating the interaction between two droplets with distinct morphologies in the Cassie, partial Cassie, and Wenzel states (Figure S14 and Discussion S2).

After the departure of Cassie droplets (Figure S14b, blue triangle without filling), no subsequent renucleation is observed, because the droplet rests on top of the cones and leaves no traces behind. In contrast, for partial Cassie droplets (Figure S14b, blue triangle half and fully filled), focal plane imaging reveals irregularly shaped wetted spots. These spots act as nucleation sites for new droplets. The newly formed droplets grow in the same locations, maintaining the overall shape of the wetted spot, indicating that water remains trapped between the cones after the first‐generation droplet departs. For more details on coalescence of droplet with distinct morphologies please refer to Figure S14 and Discussion S2.

In the case of merging of droplets with different morphologies, resulting droplet will move toward droplet that initially had stronger contact with surface. The pinning strength generally follows the order Wenzel, partial Cassie, Cassie. Partial Cassie droplets with one wetted spot were most commonly observed during scanning, although we also observed droplets with two wetted spots or enlarged wetted areas formed after merging near defects. While the evolution of these wetted regions and their transition to the Wenzel state is beyond the present scope, it may offer insight into jumping failure and the flooding phenomenon limiting jumping condensation applications [12, 32]. Combination of sparse microconical surface structures with proposed optical characterization methods provides a promising framework for future detailed studies of flooding mechanisms.

Assuming that the wetted spot represents the contact area between the surface and droplet (Figure S15a, c), we calculated the contact angle of the microdroplet and compared it with ESEM data (Figure S15d). Both methods exhibit similar trends, though optical estimates yield slightly higher angles. Notably, all samples including the non‐jumping with *p* = 4 µm spacing sample bypass the threshold 160° contact angle indicating the ability to jump after droplet coalescence. For the *p* = 4 µm sample, a partial Cassie droplet would need to reach a radius of ca. 24 µm to jump. However, after merging, the droplet transitions to the Wenzel state. Even if a partial Cassie droplet reached the critical radius without coalescence, neighboring droplets already in the Wenzel state would prevent jumping upon merging. We defined the critical jumping radius from optical data *r_c_^opt^* as the minimum radius where the contact angle exceeds 160 °, and compared it with the experimental *r_c_* and the coalescence radius $r_{e}=1/\left(4\sqrt{N_{s}}\right)$ (Figure S15d). The lower *r_c_^opt^* values relative to *r_c_* suggest potential for optimizing the jumping radius by increasing the nucleation density *N_s_*.

#### 2.3.1 Effect of Droplet's Morphology on Early Stages of Droplet Growth

Analysis of the early stages of droplet growth reveals that Cassie and partial Cassie droplets grow differently. Cassie droplet (Figure 3e) starts to grow on the top side of the cone (droplet is in focus), spreading subsequently between the tops of two (2.2 s), three (2.6 s), four (4.2 s) and finally occupying six cones arranged hexagonally (5 s). The droplet is reflecting the light during the growth, and the cones are not visible through the droplet when focused on the top of the cones indicating that the droplet is resting on the cones’ tops.

Growth of a partial Cassie droplet (Figure 3f) starts between the bottoms of the cones (the focal plane is on the tops of the cones and the droplet is out of focus), spreading and growing between four neighbouring cones (0 – 2 s). Upon reaching the critical volume, the droplet wets a hexagon with one cone inside (2.2 s). At this stage, the droplet is ‘flat’ because all cones can be seen through the droplet without optical distortions. Then the volume of the droplet starts to increase, and the droplet becomes darker (3 s). After 4.6 s of growth, the droplet starts to act as a magnifying lens, and subsequently three (4.6 s), four (5.4 s), 7 (9.2 s) and more cones (13 s) can be easily seen. When the droplet grows further (after 16.4 s), it becomes dark, and cones are no longer visible. For our system wetted area remains pinned when droplet starts to inflate upward that aligns with three‐step inflation model [14]. Although developed for nanocones, this model provides a sensible predictions for microcones, correctly capturing the trend that increased spacing favors sideways growth. Optical observations are fully confirming ESEM results on droplet growth (Figure S10e,f).

#### 2.3.2 Effect of Droplet Morphology on Individual Droplet Growth Rate

Because Cassie and partial Cassie droplets possess fundamentally different liquid–solid contact areas, their underlying morphology strongly dictates localized baseline growth dynamics. Individual growth analysis reveals that partial Cassie droplets exhibit accelerated growth rates compared to full Cassie droplets, a trend clearly captured when tracking radius evolution over time (Figure S16). This growth disparity is most pronounced on sparse microcones, but remains observable on dense cones due to the intrinsically higher solid‐liquid contact area of the partial Cassie state, providing a lower thermal resistance pathway for latent heat dissipation. An overview of individual growth trajectories across all geometries (Figure S17) shows that dense arrays (*p* ≤ 1.5 µm) yield nearly identical, suppressed growth rates, whereas on sparse structures (*p* = 2 – 3 µm), the growth rate increases proportionally with cone spacing. This behavior stems from a coupled combination of structural and transport effects. In the presence of non‐condensable gases (NCGs), the high nucleation density and close inter‐droplet proximity on dense surfaces generate overlapping mass‐diffusion boundary layers. This local vapor depletion suppresses individual droplet growth. Conversely, droplets on sparse arrays are further apart, remaining spatially isolated longer and avoiding vapor‐depletion interactions [33]. This enables them to fully exploit uninhibited local vapor fluxes. Interestingly, a distinct inversion occurs at a spacing *p* = 4 µm. Droplets in this regime predominantly adopt the Wenzel state, yet their growth rate is notably slower than partial Cassie droplets at *p* = 3 µm spacing. We attribute this penalty to a cavity‐filling bottleneck: droplets in the Wenzel state must initially expend mass filling the interstitial microcone voids before expanding vertically and outward. In contrast, partial Cassie droplets experience only localized pinning across a limited number of structural cells before immediately transitioning into efficient vertical inflation.

#### 2.3.3 Droplet Morphology Distribution

The proportion of different droplet types (Figure 3g) was defined by focal‐plane scanning in an area of at least 0.62 mm^2^ free from defects and contamination after 30 min of the condensation experiment. On the dense cones, the Cassie state dominates. As the spacing increases, the partial Cassie state becomes dominant and still allows droplet jumping. Finally, when the spacing equals the cone height, the Wenzel state becomes dominant and droplets are no longer able to jump. For the sample with *p* = 1.5 µm spacing, the partial Cassie state is dominant, unlike for P2 µm, where both partial Cassie and Cassie states are present. Otherwise, the trend of increased dominance of the partial Cassie state with increased spacing is valid for all other samples.

### 2.4 Design Parameters of Superhydrophobic Microstructures for Different Applications

#### 2.4.1 General Considerations About Surface Design and Coating Properties

Jumping condensation is sensitive to many parameters including: (i) surface topography design [2, 3, 13, 22, 23], (ii) nucleation density [13, 21], (iii) environmental conditions [13], and others. Here we explore the former two parameters under fixed environmental conditions.

For sustained jumping condensation, droplets must remain highly mobile, in partial Cassie or full Cassie states, while avoiding transition to the Wenzel state, which prevents jumping and causes strong pinning as on a planar surface [9]. We created a geometry–wetting map derived based on condensation data of our microconical system at moderately humid conditions *S*  =  1.6 ± 0.2 in the presence of NCGs (Figure 4a) taking into account nucleation density in relation to the spacing between cones *<L>/p* and energy criterion E^*^ reflecting the geometrical aspect of the system [21]. The region where Cassie and partial Cassie morphologies dominate lies within the parameter space defined by E^*^ ∈ [0, 1] and *<L>*/*p* ≥ 10.

**FIGURE 4:**
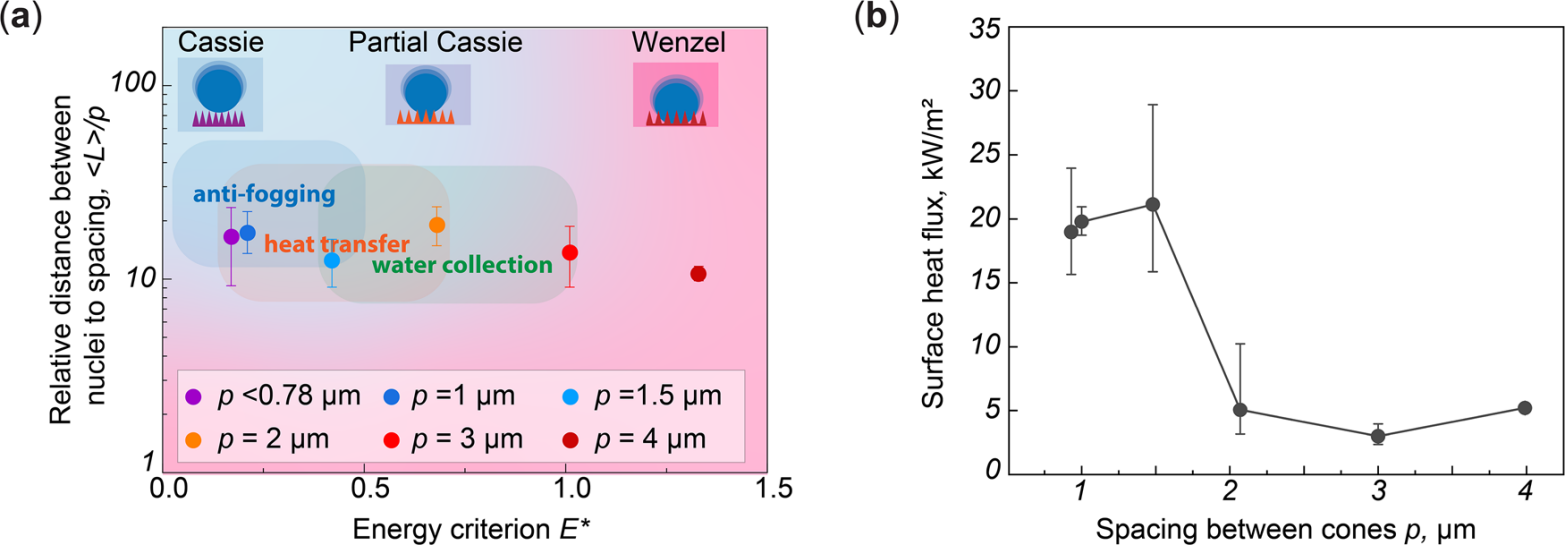
Implications of droplet morphologies for different applications (a) Surface design suggestions based on energy criterion E^*^ and nucleation density in relation to spacing between cones *<L>/p* resulting in different droplet morphology; (b) Predicted surface heat flux in a vacuum chamber as a function of cone spacing *p* using a physics‐based thermal resistance model. The model accounts for interfacial heat transfer, droplet conduction, coating resistance, and morphology‐dependent contact (vapor gap for Cassie, finite contact area for partial Cassie and Wenzel). Error bars on a, b are standard deviations calculated from at least 4 measurements (*n* = 4…20).

Along with E^*^, the nucleation density, and thus *<L>*/*p*, governs both the Cassie–Wenzel transition [21, 23] and the jumping dynamics. The stable Cassie region is characterized by *<L>*/*p* ≥ 10, while the Wenzel region is observed at *<L>*/*p* ≈ 1 due to defects (in Figure S18a, *<L>*/*p* ≈ 3.3; *<L>*/*p* ≈ 1 is used here as a simplified characteristic value; these data were excluded from the condensation analysis). Jumping optimization can be achieved through optimizing nucleation density: *<L>*/*p* > 10 is associated with slower jumping dynamics and larger critical jumping radius (Figure S18b, d), while *<L>*/*p* ≈ 10 yields more frequent jumps and optimal jumping radius (Figure S18c,d). Exploring different environmental conditions is beyond the scope of the present work, however, we hypothesize that until *<L>*/*p* ≥ 10 is satisfied, we expect similar jumping efficiency regardless of supersaturation level [3]. However, systematic investigation of different levels of supersaturation and the presence of NCGs could provide further insights into the boundaries of jumping condensation.

Within the range 0 < *E* ^*^ < 1, increasing E^*^ generally promotes a transition from Cassie‐dominated to partial Cassie morphologies, followed by a transition to the Wenzel state at E^*^ > 1. However, this trend can be shifted, likely due to nanoscale defects or contaminants, as evidenced by the prevalence of partial Cassie droplets on samples with *p* = 1.5 µm spacing (Figure 3g) and the unexpected dominance of Cassie droplets on the sample with *p* = 2 µm (Figure S18c). When defects are mainly located on the tops of the cones, nucleation occurs there, leading to the formation of Cassie droplets (Figure 3e). In contrast, defects at the base of the cones would promote nucleation at the base, resulting in partial Cassie droplets (Figure 3f). It should be noted that such an approach is effective when hydrophobic coating is uniform and localized defects are sparse enough (*<L>*/*p* ≥ 10).

The relationship between coating uniformity and nucleation density in jumping condensation has not yet been fully established [13, 34]. Contact angle hysteresis on planar surfaces is associated with chemical heterogeneities or/and roughness [35]. Low hysteresis is associated with uniform and smooth coating resulting in uniformly distributed and reduced nucleation density *N_s_* (on a planar surface) [25]. Although low hysteresis on planar surfaces does not ensure low nucleation density on microstructures (Figure S19), we suggest to optimize the coating's deposition procedure for sustained jumping condensation [36, 37, 38].

#### 2.4.2 Anti‐Fogging Applications

Anti‐fogging refers to minimizing water condensation on surfaces cooled below the dew point in humid environments. Traditionally, a hydrophilic coating is used to reduce light scattering by forming a smooth, continuous water layer instead of discrete droplets [39]. However, this approach has drawbacks: (i) variable water film thickness lowers optical measurement accuracy, and (ii) coatings are easily contaminated. Alternatively, superhydrophobic surfaces exhibiting jumping condensation could repel condensing droplets limiting the amount of water on the surface and droplet size [2, 3]. Transparent anti‐fogging surfaces can be achieved with nanoscale roughness that doesn't scatter visible light, while applications not requiring high level of transparency (e.g., infrared sensors, radio equipment, electronic enclosures) can use microstructured surfaces instead.

Anti‐fogging based on jumping condensation strategies involve minimized nucleation density, surface coverage *Φ*, and hence low amount of present condensed water on the surface *V*, minimal average droplet radius <*R>*, high jumping rate *J* and number of jumps *N_j_*. In terms of droplet morphology, Cassie droplets are preferable for anti‐fogging applications since they do not renucleate after departure (Figure 4b) and have a slower droplet radius growth rate compared to partial Cassie droplets (FigureS16). Similar considerations about anti‐fogging can be applied to anti‐icing applications; however, we acknowledge that this application is more challenging. During the freezing of condensing droplets, low surface coverage is preferred, and the dominance of Cassie droplets is desirable, as they can be easily removed from the surface and prevent the formation of ice bridging, which is a  major limitation of superhydrophobic surfaces for anti‐icing applications [40, 41, 42, 43].

In terms of surface geometry, our experimental results suggest that surfaces with dense features (E^*^ ≪ 1 in our conditions) may provide a useful candidate for future experimental evaluation of anti‐fogging performance. Nucleation density needs to be optimized in the range of *<L>/p* ≥ 10 according to the application, since it will affect jumping dynamics. With very low nucleation density, a smaller number of droplets will be generated, but it will come with the cost of slower jumping dynamics and larger critical jumping droplet radius *r_c_* (see discussion about Figure S15). Local nanoscale defects on the top of the structures may alter the proportion of condensing droplets toward the Cassie state.

#### 2.4.3 Heat Transfer

Jumping condensation enhances heat transfer by continuously refreshing the surface through droplet departure and renucleation, shifting the time‐averaged droplet size distribution toward smaller radii and lowering the overall thermal resistance from vapor to substrate. Maximizing this performance requires a sustained high nucleation site density, characterized by *<L>*/*p* ≈ 10, and promoting both a high jumping rate *J* and total number of jumps *N_j_* [44].

However, active droplet removal alone does not guarantee high thermal performance. The heat flux reflects a competition between the per‐droplet resistance, which depends on droplet morphology, and the population sustained by renewal. To examine this competition, we combined individual droplet resistances with population dynamics (Discussion S3 and Figure S20), incorporating interfacial, droplet conduction, coating, and morphology‐dependent substrate resistances [34, 45]. Three inputs are taken from measurement: the solid fraction, obtained from the measured cone tip diameter and spacing (Table S1); the departure radius of jumping droplets, taken from the measured critical jumping radius *r*_c_ rather than the predicted coalescence radius *r*_e_ (both in Figure S15e); and the droplet morphology proportions from Figure 3g.

The calculation is performed for pure vapor. In the present experiments, the condensation heat flux obtained from the condensed volume (Figure S8) is ∼25 – 46 W/m^2^ and varies by only 1.8‐fold across the whole series, with no two surfaces differing by more than 0.9 standard deviations. In air, the non‐condensable gas resistance dominates and surface geometry cannot express itself in the measured flux [46]; the calculation is therefore evaluated in the NCG‐free limit, where the geometric contributions can be compared. It is intended as a mechanistic comparison between morphologies and spacings rather than as a prediction of achievable performance.

In this limit, the predicted heat flux (Figure 4b) increases modestly across the three densest surfaces, from 19.0 kW/m^2^ on “black silicon” to 21.1 kW/m^2^ at *p* = 1.5 µm, and then falls sharply to 5.1 and 3.0 kW/m^2^ at *p* = 2 and 3 µm. The fall is governed primarily by the departure radius, which grows from 4.2 to 25.3 µm; holding it constant removes the decline for *p* ≤ 3 µm. Droplet morphology modulates this trend at fixed geometry, where the partial Cassie mode exceeds the Cassie mode by 2.0‐fold on “black silicon” and 2.3‐fold at *p* = 1.5 µm, because liquid bridges conduct across the vapor gap beneath a Cassie droplet. The maximum falls on the *p* = 1.5 µm surface, where partial Cassie droplets are almost the sole morphology (Figure 3g); the three densest surfaces are not resolved from one another within the propagated uncertainty, whereas the subsequent decrease is. The decline does not continue to *p* = 4 µm, where the predicted flux rises to 5.2 kW/m^2^ comparable to *p* = 2 µm value. This is the only surface that is entirely Wenzel, and at this spacing a fully wetted surface transfers more heat than a partial Cassie one; the rise therefore reflects the change in morphology rather than an improvement with spacing.

Several limitations bound what can be concluded from this calculation. It returns the instantaneous flux for the measured morphology proportions and does not follow their evolution; because coalescence between partial Cassie droplets can itself trigger a Wenzel transition (Figure S14a), that population is progressively depleted. The *p* = 4 µm surface is the endpoint of this process, with no jumping (Figure S6) and gravity‐only removal, so its predicted flux is an upper bound with respect to droplet removal. These limitations mean the calculation identifies the mechanisms governing the trend rather than establishing an optimal design.

With that qualification, the results indicate that spacings sustaining a sufficiently high nucleation site density while placing droplets in the partial Cassie state are favorable, since this morphology retains solid‐liquid contact for heat transfer while preserving the mobility required for coalescence‐induced departure. Partial Cassie droplets also grow faster than Cassie droplets on the same surface (Figure S16) and, unlike them, leave a wetted spot from which a new droplet renucleates after departure (Figure S14). Stabilizing this morphology requires nucleation at the cone bases rather than at the tips, which calls for a uniform coating with sparse, localized defects.

#### 2.4.4 Water Collection

Jumping condensation can also be beneficial for water collection [11, 43]. By rational surface design, its performance can be optimized [43]. Since water collection happening in the ambient atmosphere, the presence of NCGs significantly affects condensation performance, see [43] and discussion above. The main goal is to maximize the amount of water collected from the atmosphere during the phase change. Requirements for water collection are similar to heat transfer case, however, critical jumping radius must not be too small to ensure that jumping droplet could be collected before evaporation.

Our experimental observations suggest that intermediate feature densities (approximately E^*^ ≈ 0.5 under the present conditions) promoting partial Cassie morphology may be promising candidates for future water‐collection studies. Maximum amount of condensed water (water trapped on the sample *V* and removed by jumping *V_j_*) was observed on the dense cones and *p* = 2 µm samples (Figure S8), with critical jumping radii *r_c_*, of 6.3 ± 1 vs. 14.5 ± 6.8 µm respectively (Figure 2a). These results suggest potential benefit of sparse features exhibiting jumping (*p*  =  2 µm in our conditions) for water collection purposes. Uniform coating ensures sustained water collection, preventing the transition to the Wenzel state that is associated with loss of performance.

## 3 Conclusion

This work demonstrates that microstructured surfaces can exhibit a spacing‐dependent transition that strongly influences jumping condensation. Within the investigated sample series and environmental conditions, the observed transition between efficient jumping and suppressed droplet departure, consistent with the onset of Wenzel wetting, strongly correlates with cone spacing. However, rigorous studies of cones’ geometrical parameters, such as height, base diameter, opening angle, etc. are required to fully establish the capabilities of microstructures for jumping condensation. Our results further show that macroscopic wetting characterization of superhydrophobic surfaces (advancing and receding contact angles, contact angle hysteresis, droplet adhesion forces, etc.) alone are insufficient to predict condensation behavior, as microdroplets interact with the full 3D geometry of the cones rather than only their tips.

The microconical architecture enables optical classification of Cassie, partial Cassie, and Wenzel droplets based on focal‐plane scanning. This classification is supported by complementary optical, magnification, confocal, and ESEM observations, although quantitative event‐level assignment of wetting state to individual jumping events remains to be established. Dense arrays favor Cassie droplets, while increasing spacing promotes partial Cassie states whose residual wet patches seed new nucleation. Local nanoscale defects, combined with a uniform low‐surface‐energy coating, additionally modulate these morphologies, revealing multiscale sensitivity of condensing droplets. Insights into droplet morphology and its modulation, together with nucleation density, enable manipulation of the jumping dynamics that governs macroscopic performance of those surfaces.

Under the fixed environmental conditions investigated here, the resulting geometry–wetting map provides a framework for identifying relationships among microstructure geometry, droplet morphology, and jumping dynamics. Its generality across supersaturation levels and non‐condensable‐gas concentrations remains to be established. The resulting geometry–wetting design map provides a rational pathway for the future engineering of more robust microstructured surfaces using scalable methods tailored for specific application, e.g., anti‐fogging, heat‐transfer enhancement, and water harvesting.

By employing model microconical structures with variable spacing in combination with simple optical microscopy techniques, we establish a framework for future application‐oriented research. This framework enables the exploration effects of environmental conditions (e.g., supersaturation level and presence of non‐condensable gases), detailed analysis of droplet renucleation dynamics, unraveling flooding mechanisms responsible for the gradual degradation of jumping performance, and detailed investigation of how nanoscale defects or coating heterogeneities can be exploited to tailor droplet morphology and condensation dynamics.

## 4 Experimental Section

Details of all experiments are included in the Supporting Information.

## Author Contributions

M.S.K. designed the experiments and performed optical imaging of condensation including focal‐plane scanning; SEM, ESEM. Optical imaging data were analyzed by M.S.K. with the help of M.L.; T.K. developed and validated the magnification effect model, analyzed optical focal‐plane scanning and performed confocal imaging. T.Kos. (**Tomi Koskinen**), R.R. and V.P.J. fabricated microstructured surfaces, which were coated by M.S.K. with aid of P.K. Wetting characterization was performed by M.S.K. and P.K. (CA of planar samples), S.L. (CA of microstructured samples), and M.V. (SDAM); J.L and N.M. contributed to heat transfer modelling. R.H.A.R., J.V.I.T., I.T. supervised experiments and sample preparation. M.S.K. prepared the original draft of the manuscript; all authors reviewed and approved the manuscript.

## Funding

Finnish Research Impact Foundation (Vaikuttavuussäätiö) project No. 239 (R.H.A.R, M.S.K.), Business Finland grants No. 5496/31/2024 (R.H.A.R, M.S.K.) and 5714321(M.V.); Research Council of Finland projects No. 364204, 346109, 342169 (R.H.A.R.), 341459 (V.J.), 364206, 346112 (J.V.I.T.), 361541 (M.V.); International Institute for Carbon Neutral Energy Research (WPI‐I2CNER) (N.M.).

## Conflicts of Interest

The authors declare no conflicts of interest.

## Supporting information




**Supporting File 1**: advs78273‐sup‐0001‐SuppMat.pdf.

**Supporting File 2**: advs78273‐sup‐0002‐Video1.mp4.

## Data Availability

The data that support the findings of this study are available from the corresponding authors upon reasonable request.
