# Supplementary material for: Unlocking the Full Potential of Jumping Condensation on Microstructured Surfaces
Source: Adv Sci (Weinh). 2026 Oct 14:e78273. Online ahead of print. doi: 10.1002/advs.78273 (PMC13669210; doi:10.1002/advs.78273)
Supplement: Supplementary file 1 [file ADVS-9999-e78273-s001.pdf]

Supporting Information

Unlocking the full potential of jumping condensation on microstructured surfaces

Mariia S. Kiseleva[1,2], Tytti Kärki[1,2], Mika Latikka[1,2], Parham Koochak[1,2], Sakari Lepikko[1,2], Maja Vuckovac[1,2], Tomi Koskinen[3], Ramesh Raju[3], Ville P. Jokinen[4], Jiazheng Liu[5], Nenad Miljkovic[5,6,7,8,9,10], Jaakko V.I. Timonen[1,2], Ilkka Tittonen[3], and Robin H. A. Ras[1,2*]*

1. Department of Applied Physics, Aalto University, 02150 Espoo, Finland
2. Centre of Excellence in Life-Inspired Hybrid Materials (LIBER), 02150 Espoo, Finland
3. Department of Electronics and Nanoengineering, Aalto University, 02150 Espoo, Finland
4. Department of Chemistry and Materials Science, Aalto University, 02150 Espoo, Finland
5. Department of Mechanical Science and Engineering, The Grainger College of Engineering, University of Illinois Urbana-Champaign, Urbana, IL 61801 USA
6. Materials Research Laboratory, University of Illinois Urbana-Champaign, Urbana, IL 61801, USA
7. Department of Electrical and Computer Engineering, University of Illinois Urbana–Champaign, Urbana, IL 61801, USA
8. International Institute for Carbon Neutral Energy Research (WPI-I2CNER), Kyushu University, 744 Motooka, Nishi-ku, Fukuoka, 819-0395, Japan
9. Air Conditioning and Refrigeration Center, Department of Mechanical Science and Engineering, University of Illinois Urbana-Champaign, Urbana, IL, USA
10. Institute for Sustainability, Energy, and Environment (iSEE), University of Illinois, Urbana, IL 61801, USA

*Corresponding authors: mariia.kiseleva@aalto.fi, robin.ras@aalto.fi

This file includes:

Experimental Section

- Note 1. Sample preparation
- Note 2. Contact angle measurements
- Note 3. Scanning droplet adhesion microscopy (SDAM)
- Note 4. Optical imaging of condensation process
- Note 5. Image processing and data analysis
- Note 6. Electron microscopy
- Note 7. Confocal laser scanning microscopy (CLSM)

Discussions

Discussion 1. Magnification analysis of condensing droplets

Discussion 2. Coalescence scenarios of droplets with different morphologies

Discussion 3. Heat transfer modelling for microconical surfaces

Tables

Table 1. Parameters of microconical surfaces

Table 2. Nucleation density statistics of condensation data

Figures S1-S20

Videos

Video 1. Condensation process on microcones with variable spacing

References

Experimental Section

Note 1. **Sample preparation**

Periodic array of microcones with variable spacing

The microcones were fabricated on undoped (100) silicon by combining electron beam lithography (EBL) and cryogenic inductively coupled plasma reactive ion etching (ICP-RIE).[1] A layer of EBL resist (AR-P 6200) was spin coated on a 100 mm wafer, after which a 3×3 mm array of disks with 80 nm nominal diameter and period of 1, 1.5, 2, 3, and 4 μm was patterned using EBL (EPBG5000pES, Vistec). After resist development, a nominal 20 nm aluminum oxide film was deposited by e-beam evaporation (Instrumentti Mattila), after which a lift-off in remover (AR 600-71, Allresist GmbH) was carried out to define aluminum oxide disks on the silicon to act as etch masks for the ICP-RIE step. The wafers were then etched in the ICP-RIE system (Estrelas 100, Oxford Instruments) using a SF_6/O_2 plasma at a temperature of -110 °C using "black silicon" method. After etching, the wafers were coated with photoresist, diced into 15×15 mm² chips, and then had the resist removed. As the result of the microfabrication process, we obtained 15×15 mm samples with conical arrays patterned over 3×3 mm region.

"Black silicon"

The etched "black silicon" (bSi) samples were fabricated by maskless cryogenic deep reactive ion etching [2] (Oxford Plasmalab System 100, Oxford Instruments). Etching parameters were as follows: SF_6 gas flow 40 sccm, the O_2 gas flow 18 sccm, and the forward power 6 W. The ICP power was 1000 W, the etching temperature −110 °C, the pressure 10 mTorr, and the etching time 7 min.

Coating

All surfaces were modified *via* chemical vapor deposition of 1H,1H,2H,2H-perfluorooctyltrichlorosilane (PFOTS, CAS Number 78560-45-9, 97%, Sigma Aldrich)[3] in custom-built chemical vapor deposition chamber (20 cm diameter, V= 4.2 L) with N_2 inlet. Activated in oxygen plasma (Diener Electronic GmbH) for five minutes samples were placed into the chamber, evacuated to 50 mbar three times, and purged with N_2 within half an hour. Before evacuation of the chamber to 50 mbar, 0.2 mL of the PFOTS precursor was injected into an open vessel in the chamber. After half an hour-long exposure to precursor vapors, the precursor vessel was removed, and the chamber was evacuated again to 50 mbar for another half an hour. Functionalized samples were left to equilibrate with the ambient air environment (T = 20 °C, humidity below 30 %) for at least 1 day before testing.

Note 2. **Contact angle measurements**

Contact angle measurements on planar surfaces

The advancing and receding contact angles on planar hydrophobic surfaces were measured and analyzed using Biolin Theta goniometer with OneAttension (v. 3.2) software using ultrapure water type 1. Using adapted protocol,[4] we first deposited a 3 µL droplet on the surface and then added an additional 2 µL at a low rate of 0.05 µL/s. Subsequently, a further 7 µL was added to the droplet at the same rate to record the advancing contact angle. To measure the receding contact angle, the droplet volume was first increased by 7 µL and then reduced by 2 µL at 0.1 µL/s, followed by another 2 µL reduction at a low rate of 0.05 µL/s to shift the droplet to the receding state. Finally, 7 µL was withdrawn at the same low rate to record the receding contact angle. The measurements were repeated at least three times on each sample. Contact angles were determined from the recorded videos using Young-Laplace fit.

Contact angle measurements on microstructured surfaces

Using the same equipment and software, the measurement protocol for microconical samples was adapted from an earlier work[4]. First, a 5 μL droplet was deposited on the test surface. Then, additional 1 μL was added to the droplet with a low rate of 0.05 μL/s to ensure that the droplet contact angle reaches the advancing state with minimized dynamic effects of the moving baseline. After that, a further 6 μL was added to the droplet at the slow rate of 0.05 μL/s to record advancing contact angle. After the 6 μL had been added, the droplet volume was first increased by 3 μL and then reduced by 3 μL to shift the droplet baseline from advancing state to receding state. Finally, a 6 μL was withdrawn with the slow rate of 0.05 μL/s from the droplet to record the receding contact angle. The measurements were repeated three times on each sample.

Contact angles were determined from the recorded videos using the polynomial fit of the OneAttension software. The Young-Laplace fit was found to be influenced by the needle immersed in the droplets; therefore, it was not used for these superhydrophobic surfaces. First, a baseline was manually placed to be at the narrowest point between the droplet and its reflection from the surface (**Figure S2a**). The same baseline level was used for both advancing and receding measurements. Then, the polynomial fit was performed to the droplet profile, and the formed contact angle was read from the intersection of the fit and the baseline. The fitting was performed for all the recorded frames, and mean values were calculated to obtain advancing and receding contact angles, see (**Figure S2b**).

Note 3. **Scanning droplet adhesion microscopy (SDAM)**

Droplet adhesion on conical surfaces was measured using a scanning droplet adhesion microscope (SDAM) [5,6]. A metal cantilever with a flexural spring constant of $k_x = 0.083$ N/m and a 1 mm SU-

8 disk holding a 1.4 mm droplet (1.5 μL) acted as the force sensor. During a measurement, the sample approached the droplet at a controlled speed (5 μm/s), made contact, and was compressed to a maximum normal force F_N= 0.5 μN. Retraction then occurred at a controlled speed (10 μm/s) until the droplet detached. Adhesion (pull-off) force was defined as the maximum force required to separate the droplet from the surface. Measurements were performed at five different locations along each sample (a line scan with a 1 mm spatial resolution).

Note 4. **Optical imaging of condensation process**

Experimental setup

Optical imaging of breath figures on microconical structures was performed using a custom-built setup (**Figure S3**). The condensation process on microstructured surfaces was induced by cooling the sample using precision liquid nitrogen cooling stage (HFS91, Linkam) in controlled environment provided by custom-made flow-through humidity chamber with typical flow around 1.8 Lm^{-1}. We used inverted portable microscope (Mitutoyo) with long-working-distance 20× objective (PLAN APO, Mitutoyo) equipped with 5 MP camera (Ximea) providing image size 430×358.8 μm (2464×2056 pixels). Air temperature and humidity in the vicinity of the sample was recorded every second by calibrated temperature and moisture meter (635-2, Testo) equipped with data logger (Comfort Software, Testo).

Experimental procedure

Samples were placed upside down and cooled below the dew point from room temperature at a cooling rate 5°C/min, reaching a supersaturation level S = 1.6±0.2 under the following ambient conditions: humidity RH = 45±2 %, sample temperature T_s = 1.62±0.18 °C, air temperature T_{air} =

20.9±0.5 °C. Chosen supersaturation level S = 1.6±0.2 can be considered as a highly humid condition which can be found in nature and industry processes. The condensation process was recorded with 1-5 fps within 30 minutes after reaching a dew point (S = 1). Experimental conditions (RH, T_s, T_{air}) were kept constant across experiments, although fluctuations in T_{air} occurred due to convective cooling during the experiment. However, this trend was typical for all samples. Recordings were made at defect-free spot on the sample that was purged with pure nitrogen before every experiment. Upon finishing the recording, at least 5 single images were taken in random spots of the sample within 3×3 mm area. When the sample was taken from the chamber after the experiment, a 5× magnification image was taken to ensure uniform distribution of droplets across the imaging area caused by defects, contamination or heating from objective.

Note 5. **Image processing and data analysis**

The image series capturing droplet condensation were processed and analyzed using custom functions written in MATLAB. The images were first processed using functions from the MATLAB's Image Processing Toolbox to extract droplet information. Each image was converted to a binary format using a user-defined intensity threshold. The resulting droplet regions were filled to enable reliable estimation of the area fraction covered by condensing droplets in each frame. To determine individual droplet sizes, a watershed transform was applied to the binarized images to separate closely spaced droplets. Droplets located partially outside the image boundaries were excluded to avoid bias in the estimation of individual droplet properties. The boundaries of the remaining droplets were then traced and fitted with ellipses, from which the droplet coordinates, projected areas, and major and minor axis lengths were extracted. Given the large number of high-resolution images recorded, the image processing was parallelized with the Parallel Computing Toolbox to improve performance.

After image processing, the extracted data were analyzed to track individual droplets and identify jumping and merging events. The droplet data were reorganized so that each individual droplet was assigned a unique identifier that remained constant throughout the image series. This was achieved by matching droplets between consecutive frames based on spatial proximity, assuming that droplet movement between frames was minimal. Image noise and small variations in illumination occasionally led to identification errors, such as false detections of unrealistically large droplets appearing between frames. These artifacts were identified and removed from the dataset.

Droplet jumping and merging events were detected by identifying droplets that disappeared between consecutive frames or exhibited a sudden increase in radius. These droplets were then grouped according to their spatial proximity to classify events involving multiple droplets. Finally, the extracted droplet data were processed and visualized using a series of custom MATLAB functions.

Note 6. **Electron microscopy imaging**

Scanning Electron microscopy imaging (SEM)

Scanning electron microscopy was performed with Sigma VP (Zeiss) microscope. Iridium coating of 4 nm thickness was sputtered (Leica EM ACE600) to minimize charging of the surface. Imaging was performed using the In-lens detector with beam energy of 2-3 keV.

Environmental Scanning Electron microscopy imaging (ESEM)

The dynamics of water condensation at the microscale was imaged using environmental scanning electron microscope (Quanta 250, FEI) equipped with GSED detector. To reduce radiation damage and surface heating a low beam energy 5-7 keV, and 4.8 mm working distance were used. The

sample was mounted vertically on custom-made Peltier cooling stage tilted for 10-15° to achieve sample tilting 75-80°. Five purging cycles with pressure varying between 150 and 600 Pa were performed before every experiment for removing non-condensable gas from the chamber. After purging the sample was cooled to 0°C (the whole chamber was cooled down to 5°C) at a vapor pressure of 200 Pa. Water condensation is later achieved by gradual increase of the chamber pressure to 630-675 Pa. The corresponding images are saved every 0.2 s for a few minutes.

Contact angles were extracted from ESEM images by measuring apparent contact area of the droplet with the surface d and droplet radius R (**Figure S15a-b**). By applying simple geometrical relations, we obtained contact angle $\theta = \frac{\pi}{2} + \arccos(d/\mathrm{R})$.[7] This procedure was repeated for consecutive series of frames to capture contact angle dynamics.

Note 7. **Confocal laser scanning microscopy (CLSM)**

The magnification effect of microscopic lenses was verified with reflection interference contrast microscopy (RICM) using confocal microscopy (Zeiss LSM710 Axio Examiner) with 20× objective (Zeiss LD Plan-Neofluar 20x/0.4 Korr). Ball lenses N-BK7 with 300 and 500 µm diameters (Edmund Optics, 45-552, 45-553) were used for method verification.

Discussion 1. **Magnification analysis of condensing droplets using optical microscopy with variable focus**

The lens effect of microscopic condensing droplets on superhydrophobic microcone surfaces

The spherical shape of the condensing microscopic droplets on superhydrophobic surfaces enabled them to act as microscale ball lenses, magnifying the surface they are sitting on.[8,9] The spherical shape is maintained due to the absence of external forces that would otherwise deform the droplets. Firstly, the size is substantially smaller than the capillary length ($R \ll \lambda_\mathrm{c}$, where $\lambda_\mathrm{c} = \sqrt{\gamma/\rho g}$, where γ is surface tension, ρ is density of the liquid and g is the gravitational acceleration). Therefore, gravity does not deform these droplets unlike larger droplets ranging from millimetres to centimetre in size. Secondly, the ESEM images (**Figure 3b-c,** and **Figure S10**) support that droplets on present microcones are nearly spherical on both dense and sparce cones up with nearly perfect constant curvature, making them suitable for lens applications with simple optical geometry.

The optical geometry varies depending on droplet's wetting state, resulting in different magnification effects on the microcone surface they sit on (**Figure S11**). We begin by considering the lens equation for any curved surface, derived from Snell's law of light refraction: $\frac{n_1}{s_\mathrm{o}} + \frac{n_2}{s_\mathrm{i}} = \frac{n_2 - n_1}{R}$ (Eq. 1), where n_1 is the refractive index (RI) of the medium where the incident ray travels, and n_2 is the RI of the medium where the refracted ray travels, s_o is object distance and s_i is image distance, and R is the radius of curvature of the interface between the two mediums [10]. Both Wenzel and Cassie droplets create an image below the droplet (s_i <0) (**Figure 11a-b**). The magnification M is given by $M = -\frac{s_\mathrm{i}}{s_\mathrm{o}}$. Here, the negative s_i results in positive magnification, indicating that both Wenzel and Cassie droplets magnify the surface underneath. However, as

Cassie droplets refract light at both the top and bottom interfaces, the image forms further below, enhancing the magnification effect (**Figure S11b**). Thus, when observing textures surfaces through microscopic droplets in Wenzel and Cassie states, differences in magnifications are evident (**Figure S11c**). By solving image distance with Eq. 1: $s_\mathrm{i} = \frac{n_2 R s_\mathrm{o}}{n_2 s_\mathrm{o} - n_1 (s_\mathrm{o} + R)}$, we calculated the expected magnification effect of drops with different radius *R* and wetting state and compared to experimentally measured ones.

To verify the model, we utilized solid ball lenses placed on microcone surfaces and measured their magnification factors (**Figure S12a**). The stability and good optical properties of the ball lenses enables us to obtain high-resolution confocal *z*-stack through the ball lens. This allowed us to verify, firstly, that the calculated image distance s_i matches the experimentally measured one, and, secondly, that the model magnification M_model complies with the measured experimental magnification factor M_exp. The glass ball lens (*R* = 250 µm) magnified the microcone surface (*p* = 2 µm) (**Figure S12b-c**). The measured experimental image distance ($s_\mathrm{i,exp} = -1064$ µm) and magnified pitch length ($p_\mathrm{mag,exp} = 6.6$ µm) were close to theoretical estimations ($s_\mathrm{i,model} = -1076$ µm; and $p_\mathrm{mag,model} = 6.5$ µm). Comparing the p_mag with real *p*, gives magnification of $M \approx 3.3$. These measurements indicate that we can model the magnifying effect of small beads on microcone surfaces and estimate whether they are in contact with the solid surface or if there is an air gap in between.

Analysis of droplet magnification factor

RICM was used to acquire images of condensation droplets on microcone surfaces. By changing the focus, three *z*-planes were imaged: the first plane, z_1, focuses on the drop middle part from where drop radius *R* was measure, the second, z_2, focuses on the dry microcone surface from which

cone pitch p was measured, and the third, z_3, is the plane for the magnified view through the droplet from which magnified cone pitch p_{mag} was measured (**Figure S13a-c**). The experimental total through-droplet imaging area A_{mag} in the z_3-plane was ~20% of the total drop area A measured from z_1-plane (**Figure S13d**), limiting the p_{mag} measurement to droplets with $R \geq 10$ µm. As the droplet curvature causes aberration (**Figure S13c**), the p_{mag} was always measured from as close to the centre of the droplet as possible. If a contact region was present in the droplet (as seen in center of **Figure S13c**), the p_{mag} was measured next to this contact region.

The measurement of R was done with line tool in ImageJ, while p and p_{mag} were measured using line and plot profile tools in ImageJ to obtain the intensity as a function of distance, and this data was further processed using a custom MATLAB code to measure the intensity peak-to-peak distance which corresponds to the distance between the microcones, thus the pitch p (**Figure S13e**). The intensity plots from different z-planes were also used to validate the correct focus plane for both p and p_{mag} measurement. The correct z-planes were chosen according to the largest $I_{\mathrm{max}} - I_{\mathrm{min}}$ value that indicates the largest contrast between the microcone centre (dark) and the flat region between the cones (light). Three measurements and their standard deviations for p and p_{mag} were used to calculate the experimental magnification factor $M = p_{mag} / p$.

Discussion 2. **Coalescence scenarios of droplets with different morphologies**

For further validation of droplet-state identification, we combined focal-plane scanning over an area of at least 0.62 mm² free of defects and contamination after 30 min of condensation with monitoring the events following coalescence, namely jumping or merging. For jumping droplets, we further distinguished between clean departure from the surface and departure followed by renucleation at the same location. Typical coalescence scenarios for two droplets in different states are presented in the diagram. (**Figure S14a)**.

On the diagram scenarios marked with blue symbols are typical for all samples exhibiting jumping condensation ("black silicon", cones with 1, 1.5, 2, 3 µm spacing). After the departure of Cassie droplets (**Figure S14b**, blue triangle without filling), no subsequent renucleation is observed, because the droplet rests on top of the cones and leaves no traces behind. In contrast, for partial Cassie droplets (**Figure S14b**, blue triangle half and fully filled), focal plane imaging reveals irregularly shaped wetted spots. These spots act as nucleation sites for new droplets. The newly formed droplets grow in the same locations, maintaining the overall shape of the wetted spot, indicating that water remains trapped between the cones after the first-generation droplet departs.

In most cases, coalescence of Cassie and partial Cassie droplets result in jumping. However, droplets sometimes merge without jumping, particularly near strong pinning defects or when there is a large size mismatch between coalescing droplets.[11,12] In such cases, the partial Cassie droplet remains in its place absorbing the Cassie droplet (**Figure S14b**, blue circle without filling), which is not surprising for Cassie droplets being in contact with only cone tops. Merging of two partial Cassie droplets may result in absorbing one droplet (**Figure S14b**, blue circle fully filled), usually the smaller one with another, or alternatively forming one partial Cassie droplet with two wetted spots (**Figure S14b,** blue circle half filled). The latter case was observed during the merging of

two small partial Cassie droplets located close to each other. Interestingly, such droplets with 2 wetted spots preserve the ability to jump, avoiding transitioning to Wenzel state.

For non-jumping case, cones with 4 μm spacing, we observed only merging scenarios (symbols are marked in pink). At the initial stage of condensation droplets are in partial Cassie state with larger wetted spot. Although after merging two or more such partial Cassie droplets, the resulting droplet is a Wenzel droplet with an irregular shape (**Figure S14b,** pink circle half filled). Since Wenzel state is more energetically favorable, resulting droplet cannot maintain partial Cassie state anymore and collapse into more stable Wenzel configuration. When Wenzel droplet is merging with partial Cassie droplets (**Figure S14b,** pink circle fully filled) resulting in formation of Wenzel droplet as well.

In the case of merging of droplets with different morphologies, resulting droplet will move towards droplet that initially had stronger contact with surface. Wenzel droplets have strongest pinning, then partial Cassie droplets are pinned in on its wet spot, partial Cassie droplet with larger wet spot will dominate over another partial Cassie droplets with smaller spot. Cassie droplets have the weakest contact with the surface; they are almost levitating above it.

Discussion 3. **Heat transfer modelling for microconical surfaces**

The overall heat flux is calculated based on the proportions of different wetting states (**Figure 3g**), where the heat transfer for each wetting state is calculated using the specified model that combines the single-droplet heat transfer and the droplet size distribution:

$$q'' = \begin{cases} \int_{r_{\min}}^{r_{\mathrm{e}}} q(r) n_{\mathrm{C}}(r) \mathrm{d}r, & \text{Cassie state} \\ \int_{r_{\min}}^{r_{\mathrm{e}}} q(r,\theta) n_{\mathrm{PC}}(r,\theta) \mathrm{d}r, & \text{Partial Cassie state}, \\ \int_{r_{\min}}^{r_{\mathrm{e}}} q(r) n_{\mathrm{W}}(r) \mathrm{d}r + \int_{r_{\mathrm{e}}}^{r_{\max}} q(r) N(r) \mathrm{d}r, & \text{Wenzel state} \end{cases} \tag{1}$$

where q'' is the surface heat flux, $r_{\min}$ is the critical radius of embryo nucleation:

$$r_{\min} = \frac{2T_{\mathrm{sat}}\sigma_{\mathrm{water}}}{h_{\mathrm{fg}}\rho_{\mathrm{water}}\Delta T}, \tag{2}$$

in which T_{sat} is the saturation temperature corresponding to the experimental conditions, σ_{water} is the water surface tension, h_{fg} is the latent heat of vaporization of water, ρ_{water} is the water density, and ΔT is the temperature difference between the saturation temperature and the sample temperature. The term r_{e} in Eq. 1 is the coalescence radius and depends on the nucleation site density (N_{s}):

$$r_{\mathrm{e}} = \frac{1}{4\sqrt{N_{\mathrm{s}}}}, \tag{3}$$

and $r_{\max}$ refers to the departure droplet radius, where the classic model calculates it based on force analysis:

$$r_{\max} = \left[\frac{6(\cos\theta_{\mathrm{r}} - \cos\theta_{\mathrm{a}})\sin\theta_{\mathrm{e}}\sigma_{\mathrm{water}}}{\pi(2 - 3\cos\theta_{\mathrm{e}} + \cos^3\theta_{\mathrm{e}})\rho_{\mathrm{water}} g \cos\Theta} \right]^{0.5}, \tag{4}$$

where θ_{r} and θ_{a} are the receding and advancing contact angles (**Figure S1a**), θ_{e} is the apparent equilibrium contact angle and $\theta_{\mathrm{e}} = \arccos(0.5\cos\theta_{\mathrm{r}} + 0.5\cos\theta_{\mathrm{a}})$, g is the gravitational acceleration, and Θ is the surface inclination from the vertical. Rather than evaluate Eq. 4, we obtain the maximum droplet radius from the measured droplet adhesion: a pinned Wenzel droplet departs when its weight exceeds the retention force, giving $r_{\max} \approx (3F/4\pi\rho_{\mathrm{water}}g)^{1/3}$, where F is taken as an order of magnitude above the Cassie-state pull-off force measured in **Figure S1b**. This yields $r_{\max} \approx 0.5 - 0.6$ mm on the sparse surfaces where Wenzel droplets occur. The predicted flux is insensitive to this value: since Wenzel droplets appear only on the $p = 3$ and $p = 4$ μm surfaces and the small-droplet population dominates the integrated flux, varying $r_{\max}$ from 0.5 to 1.7 mm changes the result by less than 1%.

The single-droplet heat transfer rate, $q(r)$, is derived from a comprehensive thermal network that incorporates all possible thermal resistances from the ambient to the substrate: the liquid-vapor interfacial resistance due to direct vapor molecule accommodation, the resistance due to the droplet interface curvature, the conduction resistance through the droplet, the conduction resistance through the microcones and liquid bridges, and the coating resistance (**Figure S20**). Overall, the heat transfer rate through a single droplet can be written as:[13,14]

$$q(r,\theta,\Delta T) = \frac{\pi r^2 \left(\Delta T - \dfrac{2T_{\mathrm{sat}}\sigma_{\mathrm{water}}}{r h_{\mathrm{fg}}\rho_{\mathrm{water}}} \right)}{\dfrac{1}{2h_{\mathrm{i}}(1-\cos\theta_{\mathrm{e}})} + \dfrac{r\theta_{\mathrm{e}}}{4k_{\mathrm{water}}\sin\theta_{\mathrm{e}}} + \dfrac{R_{\mathrm{sub}}}{\sin^2\theta_{\mathrm{e}}}}, \tag{5}$$

where R_{sub} is the thermal resistance of the substrate:

$$R_{\mathrm{sub}} = \left[\frac{1}{R_{\mathrm{coat}}^{\mathrm{flank}} + R_{\mathrm{cone}}} + \frac{1}{R_{\mathrm{gap}} + R_{\mathrm{coat}}^{\mathrm{floor}}} \right]^{-1}, \quad (6)$$

with

$$R_{\mathrm{coat}}^{\mathrm{flank}} = \frac{\delta_{\mathrm{coat}}}{k_{\mathrm{coat}}\varphi}, \quad R_{\mathrm{coat}}^{\mathrm{floor}} = \frac{\delta_{\mathrm{coat}}}{k_{\mathrm{coat}}(1-\varphi)}, \quad (7)$$

$$R_{\mathrm{gap}} = \frac{h_{\mathrm{ave}}}{k_{\mathrm{gap}}(1-\varphi)}, \quad (8)$$

where k_{gap} is the thermal conductivity of the gap: $k_{\mathrm{gap}} = k_{\mathrm{vapor}}$ for Cassie droplets (vapor fills the gap) and $k_{\mathrm{gap}} = k_{\mathrm{water}}$ for partial Cassie and Wenzel droplets (liquid fills it); k_{coat} is the thermal conductivity of the coating; δ_{coat} is the coating thickness, h_{ave} is the average microcone height, and h_{i} is the interfacial heat transfer coefficient calculated by

$$h_{\mathrm{i}} = \frac{8\alpha}{2-0.798\alpha} \frac{\gamma-1}{\gamma+1} \frac{1}{\sqrt{2\pi R_{\mathrm{g}} T_{\mathrm{sat}}}} \frac{h_{\mathrm{fg}}{}^{2}}{v_{\mathrm{g}} T_{\mathrm{sat}}}, \quad (9)$$

in which α is the accommodation factor that refers to the ratio of vapor molecules captured by the liquid phase to the total vapor molecules reaching the liquid surface; γ is the heat capacity ratio ($\gamma = c_{\mathrm{p}}/c_{\mathrm{v}}$, where c_{p} and c_{v} are the specific heat at constant pressure and at constant volume, respectively), R_{g} is the gas constant of water vapor, and v_{g} is the specific volume of water vapor. The solid fraction φ entering the thermal resistance of the substrate is the fraction of unit cell wetted by the droplet. On microconical arrays of these dimensions, the droplet meniscus is

supported on the cone tips rather than penetrating the structure, so φ follows from the measured tip diameter d_t and the cone spacing p

$$\varphi = \frac{\pi d_{\mathrm{t}}^2}{4A_{\mathrm{cell}}}, \qquad A_{\mathrm{cell}} = \frac{\sqrt{3}}{2}p^2, \tag{10}$$

giving 0.06% – 2.7% across the series (**Table S1**) in consistence with results from [1].

The conduction resistance through the microcones is evaluated for the actual tapered geometry rather than a column of constant cross-section. A cone's cross-section grows from the wetted tip toward the base, so the conduction resistance is concentrated near the tip. Modeling each cone as a truncated cone of full opening angle β, the structure thermal resistance per cone is

$$R_{\mathrm{cone}} = \frac{A_{\mathrm{cell}}}{k_{\mathrm{struc}}\pi\tan^2(\beta/2)}\left(\frac{1}{z_{\mathrm{c}}} - \frac{1}{h_{\mathrm{ave}}}\right), \tag{11}$$

where z_{c} is the depth of the wetted contact below the virtual cone apex.[15] Because the droplet wets the cone tip, the contact patch radius is $d_{\mathrm{t}}/2$ and

$$z_{\mathrm{c}} = \frac{d_{\mathrm{t}}}{2\tan(\beta/2)}. \tag{12}$$

The wetted-contact depth ranges from z_{c} = 0.25 μm to 0.74 μm. Because d_{t} is nearly constant across the series, z_{c} is set by the tip size and the cone angle and shows no systematic dependence on spacing. The tapered treatment lowers the structure resistance by a factor of about 4 to 7 relative to a uniform column of cross-section φ, and correspondingly raises the predicted flux by 1.1- to 1.9-fold across the series (largest at the p = 1 μm).

While the substitution of k_{water} for k_{vapor} in the gap and the calculation of the wetted-contact depth z_c account for partial liquid penetration, it should be noted that the 1D parallel thermal resistance network (**Figure S20**) represents a conservative simplification. By restricting heat flow to strictly vertical parallel paths, the model inherently neglects lateral heat conduction from the partially liquid-filled gaps into the microcone flanks. Although lateral heat flow would still pass through the uniform coating, the flank surface area is much larger than the tip area; therefore, this parallel assumption yields an upper bound for the thermal resistance of penetrating morphologies.

The droplet size distribution encodes critical information on the total heat and mass transferred during condensation and is therefore essential for accurate calculations of heat transfer performance. For droplets in the Cassie and partial Cassie states, coalescence-driven jumping defines the size range of interest, spanning from the minimum nucleation radius r_{min} to the coalescence-triggered departure radius r_e. In contrast, droplets in the Wenzel state remain pinned and typically depart under gravity once reaching a size threshold governed by contact line pinning, thereby extending the upper limit to r_{max}. As a result, heat transfer contributions must be integrated differently depending on the wetting state: from r_{min} to r_e for Cassie/partial Cassie droplets, and from r_{min} to r_{max} for Wenzel droplets. Specifically, the small droplet size distribution ($r_{\text{min}} < r < r_e$) for the Wenzel state, assuming a fixed static contact angle, can be expressed as:

$$n_{\text{W}}(r) = \frac{1}{3\pi r_e^3 r_{\text{max}}} \left(\frac{r_e}{r_{\text{max}}} \right)^{-\frac{2}{3}} \frac{r\left(r_e - r_{\text{min}}\right)}{r - r_{\text{min}}} \frac{C_2 r + C_3}{C_2 r_e + C_3} \exp\left[D_1(r) + D_2(r) \right], \tag{13}$$

where:

$$C_1 = \frac{\Delta T}{\rho_{\mathrm{water}} h_{\mathrm{fg}} \left(\cos^3\theta - 3\cos\theta + 2\right)}$$
$$C_2 = \frac{\theta}{4k_{\mathrm{water}} \sin\theta}$$
$$C_3 = \frac{1}{2h_{\mathrm{i}}\left(1-\cos\theta\right)} + \frac{R_{\mathrm{sub}}}{\sin^2\theta}$$
$$D_1(r) = \frac{C_2}{C_1\tau}\left[r_{\mathrm{min}}{}^2 \ln\left(\frac{r_{\mathrm{e}} - r_{\mathrm{min}}}{r - r_{\mathrm{min}}}\right) + r_{\mathrm{min}}\left(r_{\mathrm{e}} - r\right) + \frac{r_{\mathrm{e}}{}^2 - r^2}{2}\right], \tag{14}$$
$$D_2(r) = \frac{C_3}{C_1\tau}\left[r_{\mathrm{min}} \ln\left(\frac{r_{\mathrm{e}} - r_{\mathrm{min}}}{r - r_{\mathrm{min}}}\right) + r_{\mathrm{e}} - r\right]$$
$$\tau = \frac{3r_{\mathrm{e}}{}^2\left(C_2 r_{\mathrm{e}} + C_3\right)^2}{C_1\left(11C_2 r_{\mathrm{e}}{}^2 - 14C_2 r_{\mathrm{e}} r_{\mathrm{min}} + 8C_3 r_{\mathrm{e}} - 11C_3 r_{\mathrm{min}}\right)}$$

and the large droplet size distribution ($r_{\mathrm{e}} < r < r_{\mathrm{max}}$) gives [16]:

$$N(r) = \frac{1}{3\pi r_{\mathrm{max}} r^2}\left(\frac{r}{r_{\mathrm{max}}}\right)^{-\frac{2}{3}}. \tag{15}$$

For droplets in the Cassie state, as the droplets depart by coalescence rather than shedding, the droplet size distribution gives

$$n_{\mathrm{C}}(r) = \frac{1}{3\pi r_{\mathrm{e}}^3 r_{\mathrm{max}}}\left(\frac{r_{\mathrm{e}}}{r_{\mathrm{max}}}\right)^{-\frac{2}{3}} \frac{r\left(r_{\mathrm{e}} - r_{\mathrm{min}}\right)}{r - r_{\mathrm{min}}} \frac{C_2 r + C_3}{C_2 r_{\mathrm{e}} + C_3}, \tag{16}$$

where the maximum droplet radius is given by $r_{\mathrm{max}} = r_{\mathrm{e}}$. For the jumping morphologies, the upper limit of the small-droplet distribution is the radius at which droplets leave the surface. Equation 3 gives the coalescence radius as an estimate of this radius from nucleation site density alone. Here we use instead the experimentally measured critical jumping radius r_{c} (**Figure S15e**), the directly observed departure radius rather than an estimate from nucleation site density. The two quantities

are compared in **Figure S15e**; r_c is systematically smaller than r_e on the dense surfaces and scales more steeply with spacing, so using the measured value rather than the estimate changes the predicted flux appreciably. The p = 4 µm surface does not exhibit jumping (**Figure S6**); for its Wenzel population, the coalescence radius r_e is retained as the lower integration limit, and r_{max} follows from the pinning-limited size threshold as explained near Eq. 4.

The modeled distributions assume that every jumping droplet departs on reaching r_c. Because not all coalescence events produce a jump (**Figure S6**), some droplets grow past r_c, and these are visible in the measured size distributions (**Figure S5**). Comparing the modeled population above r_c with **Figure S5** shows agreement to within a factor of about two, which supports the assumed distribution. The measured distributions are used here as a check on the model rather than as an input, since they were acquired in air whereas the calculation is performed for pure vapor.

For partial Cassie droplets, previous studies have shown that once the droplet radius becomes comparable to the structural spacing ($r \sim p$), growth proceeds with a nearly constant base area and a dynamically evolving contact angle [17]. To account for this behavior, the heat transfer rate must be modified accordingly. By relating the heat transfer rate to the droplet growth rate via latent heat and assuming a spherical cap geometry, we obtain:

$$\begin{aligned} q\left(r,\theta,\Delta T\right) = \dot{m}h_{\mathrm{fg}} = \rho_{\mathrm{water}} h_{\mathrm{fg}} \frac{\mathrm{d}V}{\mathrm{d}t} = \frac{\pi}{3}\rho_{\mathrm{water}} h_{\mathrm{fg}} \frac{\mathrm{d}}{\mathrm{d}t}\left[\left(\cos^3\theta - 3\cos\theta + 2\right)r^3\right] \\ = \pi\rho_{\mathrm{water}} h_{\mathrm{fg}} r^2 \left[r\sin\theta\left(1-\cos^2\theta\right)\frac{\mathrm{d}\theta}{\mathrm{d}t} + \left(\cos^3\theta - 3\cos\theta + 2\right)\frac{\mathrm{d}r}{\mathrm{d}t}\right], \end{aligned} \tag{17}$$

where $\dot{m}$ is the mass transfer rate and V is the droplet volume. Combining Eqs. 5 and 17, we obtain the droplet growth rate for partial Cassie droplets:

$$G_{\mathrm{PC}} = \frac{\mathrm{d}r}{\mathrm{d}t} = C_{1,\mathrm{PC}} \frac{1 - r_{\min} / r}{C_2 r + C_3}, \tag{18}$$

with the modified coefficient $C_{1,\mathrm{PC}}$ being:

$$C_{1,\mathrm{PC}} = \frac{\Delta T}{\rho_{\mathrm{water}} h_{\mathrm{fg}} \left[r \sin\theta \left(1 - \cos^2\theta\right) \frac{\mathrm{d}\theta}{\mathrm{d}r} + \left(\cos^3\theta - 3\cos\theta + 2\right) \right]}. \tag{19}$$

The dynamic contact angle for obtaining $\mathrm{d}\theta/\mathrm{d}r$ is from the fact that[13]:

$$\theta_{\mathrm{PC}}(r) = \begin{cases} \theta_{\mathrm{a}}, & r < p \\ \pi/2 + \arccos(s/r), & p < r < r_{\mathrm{CB}} \\ \theta_{\mathrm{a}}^{\mathrm{CB}}, & r \geq r_{\mathrm{CB}} \end{cases}, \tag{20}$$

where $r_{\mathrm{CB}} = p/\sin(\pi - \theta_{\mathrm{a}}^{\mathrm{CB}})$, and and $\theta_{\mathrm{a}}^{\mathrm{CB}}$ is defined as

$$\cos\theta_{\mathrm{a}}^{\mathrm{CB}} = \varphi(\cos\theta_{\mathrm{e}} + 1) - 1. \tag{21}$$

By using the population-balanced theory and applying a boundary condition as $n_{PC}(r_{\mathrm{e}}) = N(r_{\mathrm{e}})$, the droplet size distribution of partial Cassie droplets is similar to that of the Cassie droplets[13].

Tables

Table S1. Dimensions of the microconical surfaces determined from SEM.

Sample	Height *h*, µm	Cones' Ø, *d*, µm	Pitch (peak to peak), *p*, µm	Cones' tip Ø, d_t, µm	Angle *β*, °	Solid fraction, $\varphi^{1)}$, %	Surface roughness $R_f^{2)}$	Energy criterion E^*
bSi3)	4.37 ± 0.49	0.94 ± 0.17	0.93 ± 0.29	0.16 ± 0.04	12.3 ± 2.67	2.684)	8.6	0.2
1 µm	3.50 ± 0.04	0.92 ± 0.01	1.00 ± 0.04	0.10 ± 0.01	15.0 ± 0.26	0.9	6.1	0.3
1.5 µm	3.94 ± 0.03	1.39 ± 0.02	1.48 ± 0.01	0.09 ± 0.08	20.4 ± 0.29	0.34	4.8	0.4
2 µm	3.66 ± 0.03	1.38 ± 0.03	2.07 ± 0.07	0.11 ± 0.01	21.7 ± 0.46	0.26	2.8	0.7
3 µm	4.30 ± 0.02	1.40 ± 0.03	3.00 ± 0.02	0.12 ± 0.01	18.7 ± 0.44	0.15	2.0	1.0
4 µm	4.42 ± 0.01	1.34 ± 0.02	3.99 ± 0.03	0.10 ± 0.01	17.4 ± 0.28	0.06	1.6	1.3

Notes: The cones' geometry was measured from representative electron micrographs using ImageJ software. Data from at least 5 measurements (n = 5…9) measurements were averaged, and the standard deviation was calculated.

1) $\varphi = \frac{\pi d_t^2}{2\sqrt{3}p^2}$, where d_t is cone's tip diameter and *p* – pitch (spacing) distance between centers of neighboring cones.

2) the surface roughness factor $R_f = \frac{\frac{1}{2}\pi\left(\frac{d}{2}\right)\cdot\sqrt{\left(\frac{d}{2}\right)^2+h^2}+\left(\frac{\sqrt{3}p^2}{4}-\frac{1}{2}\pi\left(\frac{d}{2}\right)^2\right)}{\frac{\sqrt{3}p^2}{4}}$, where *d* is cone's diameter *h* – cones' height.

3) "black silicon" surface is stochastic irregular surface with sharp peaks of various heights having nanoscale features on top, therefore obtained values are arbitrary although they are consistent with literature [1,2].

4) Estimation of the solid fraction assuming that cones are regular and smooth, real solid fraction of "black silicon" is difficult to evaluate due to stochasticity of the roughness and presence of nanoscale features.

Table S2. Nucleation density statistics of condensation data.

**Sample**	Nucleation density N_s, mm^{-2}	Normalized nucleation density N_{3D}*, mm^{-2}
bSi	1543 ± 1122	178 ± 130
1 µm	777± 305	128 ± 50
1.5 µm	704± 341	147 ± 71
2 µm	306 ± 239	110 ± 86
3 µm	144 ± 83	71 ± 41
4 µm	136 ± 23	86 ± 14

* Normalized by the three-dimensional surface area nucleation density $N_{3D} = \frac{N_s}{R_f}$, where R_f is the roughness factor calculated in Table S1.

Figures

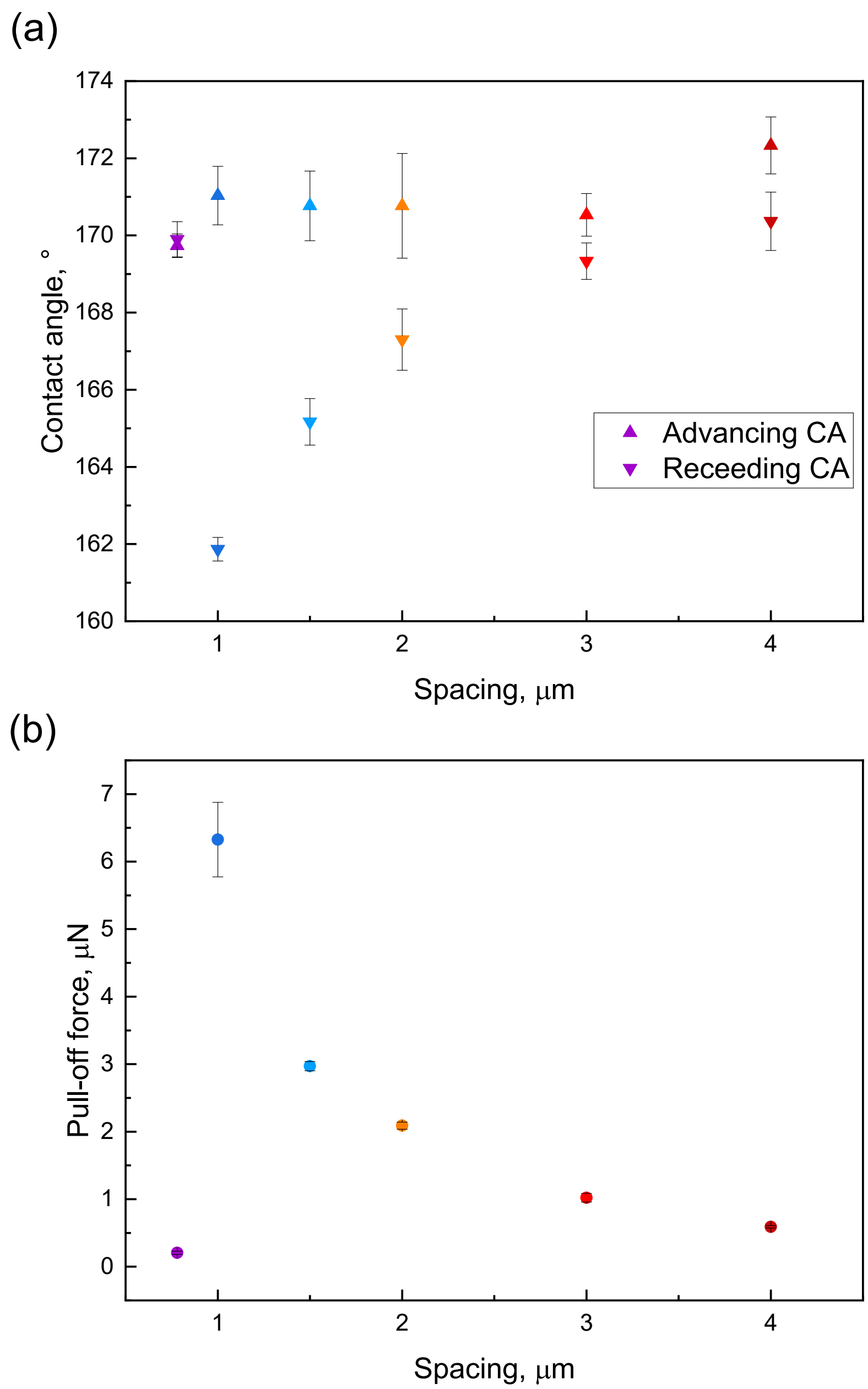

Figure S1. **Wettability measurements of conical samples**: (***a***) Advancing and receding contact measurements of three independent measurements (n = 3), (***b***) droplet adhesion measurements – pull-off force of five independent measurements (n = 5) vs. spacing p between the cones.

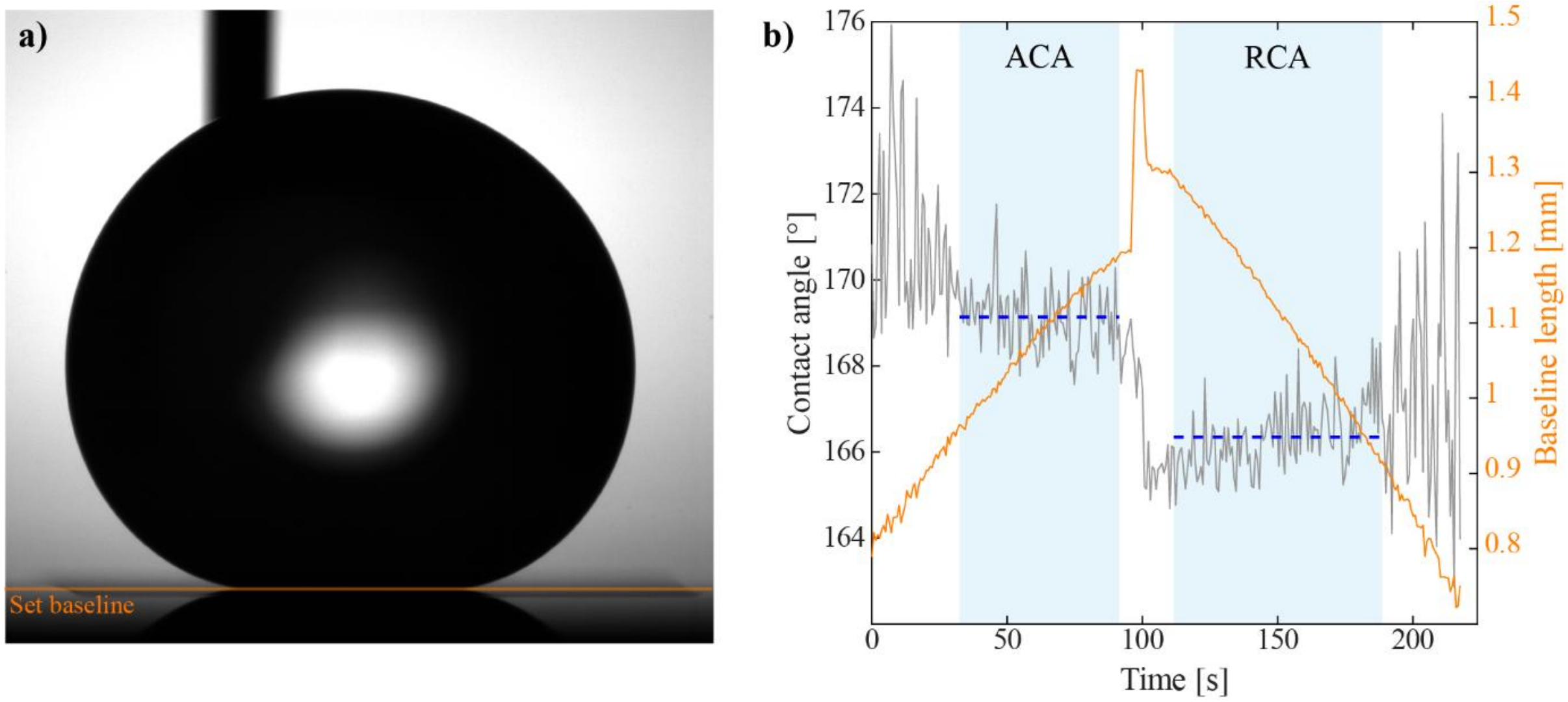

Figure S2. **Contact angle measurements on microconical surface** (***a***) A frame with a set baseline in contact angle measurements on superhydrophobic surface. (***b***) An obtained contact angle and baseline fitting from a single recording. The shaded areas represent regions over which averaging of advancing and receding contact angles were performed, and the dashed blue lines represent the obtained average values.

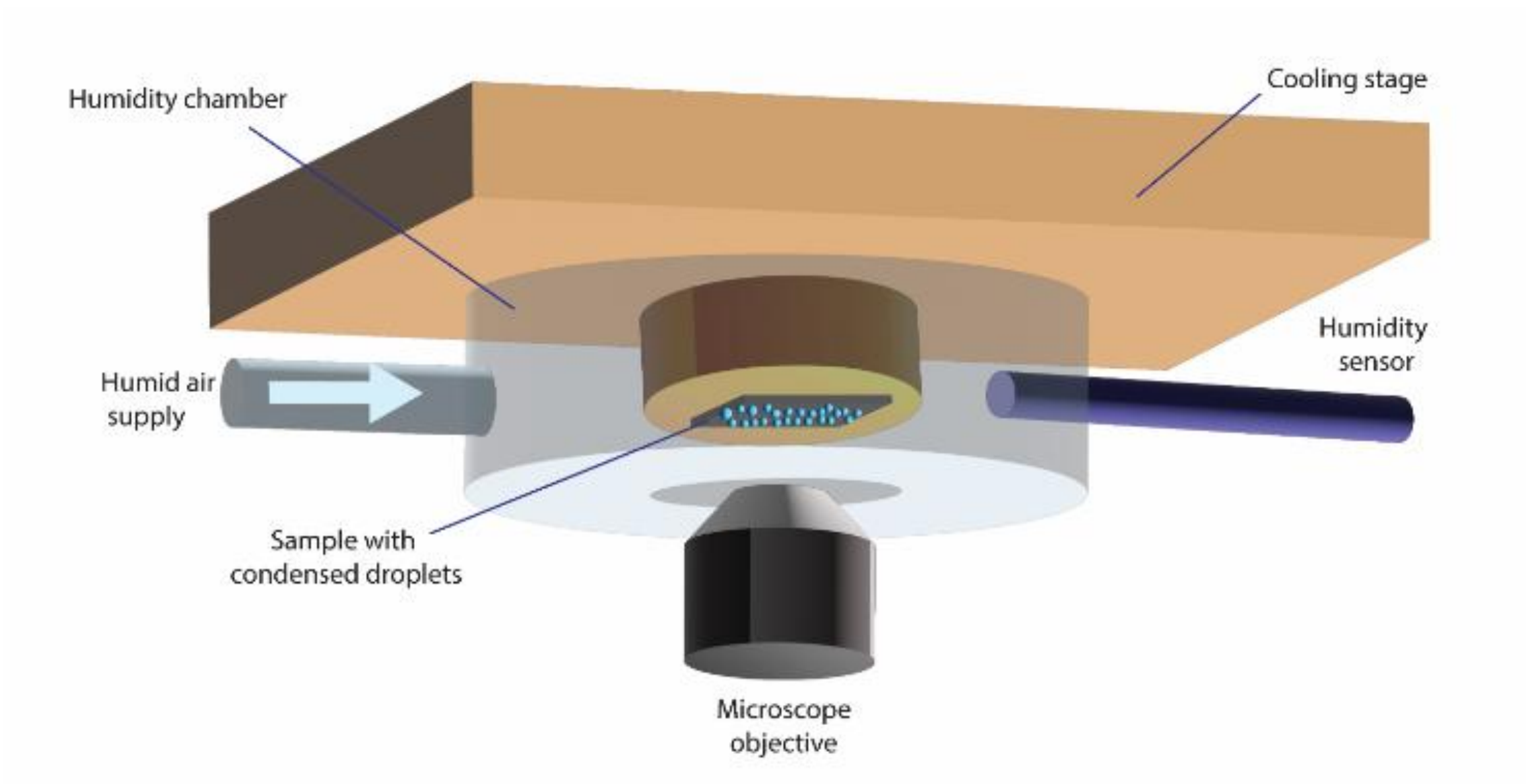

Figure S3. **Experimental setup for condensation imaging** consisting of cooling stage with attached sample, microscope with camera, environmental 'flow-through' chamber equipped with humidifier as well as temperature and humidity sensor in the proximity of the sample.

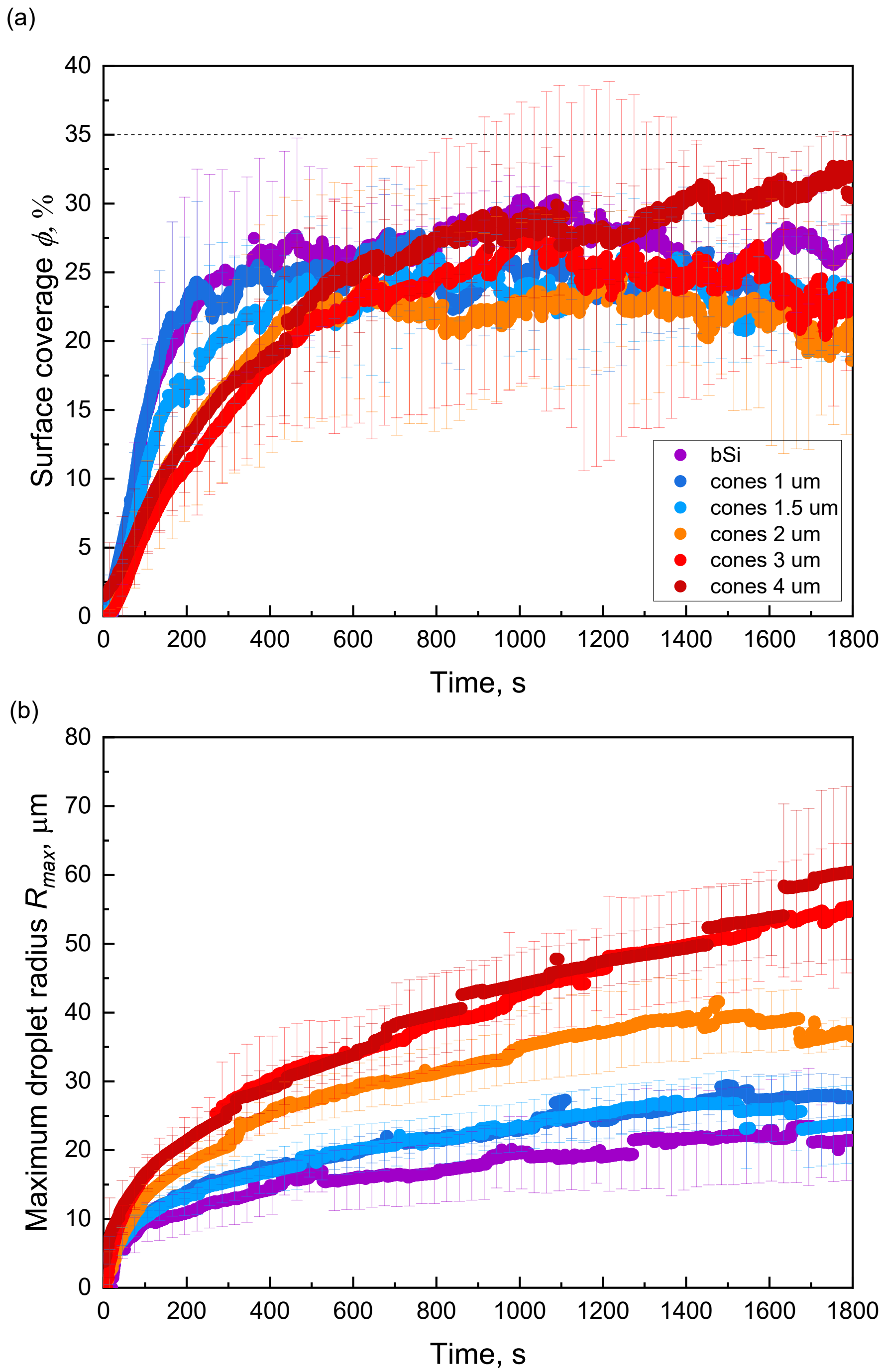

Figure S4. **Quantification of breath figures** on array of cones with variable spacing ("black silicon" and cones with 1, 1,5, 2, 3 and 4 µm spacing): (***a***) surface coverage *Φ*, (***b***) maximum droplet radius, as a function of time *t* (s). Error bars correspond to the standard deviation of at least 4 independent measurements (n = 4…5) on different samples at similar conditions.

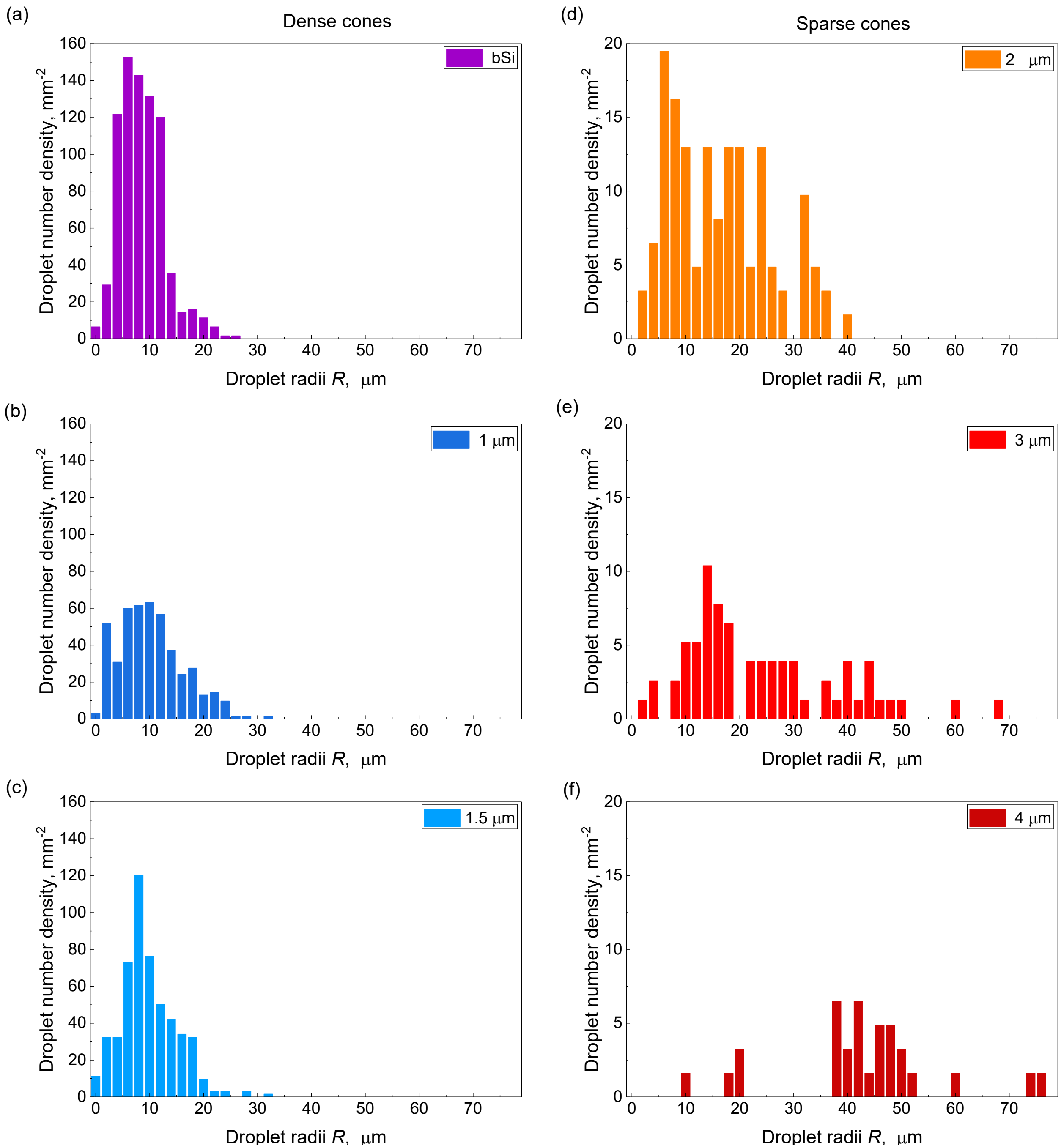

Figure S5. **Droplet size distribution** after 30 min of experiment on cone arrays with variable spacing, averaged from at least four independent measurements (n = 4…5) performed on different samples under similar conditions: (a) "black silicon" and hexagonally packed cones with spacing of (b) 1, (c) 1.5, (d) 2, (e) 3, and (f) 4 μm.

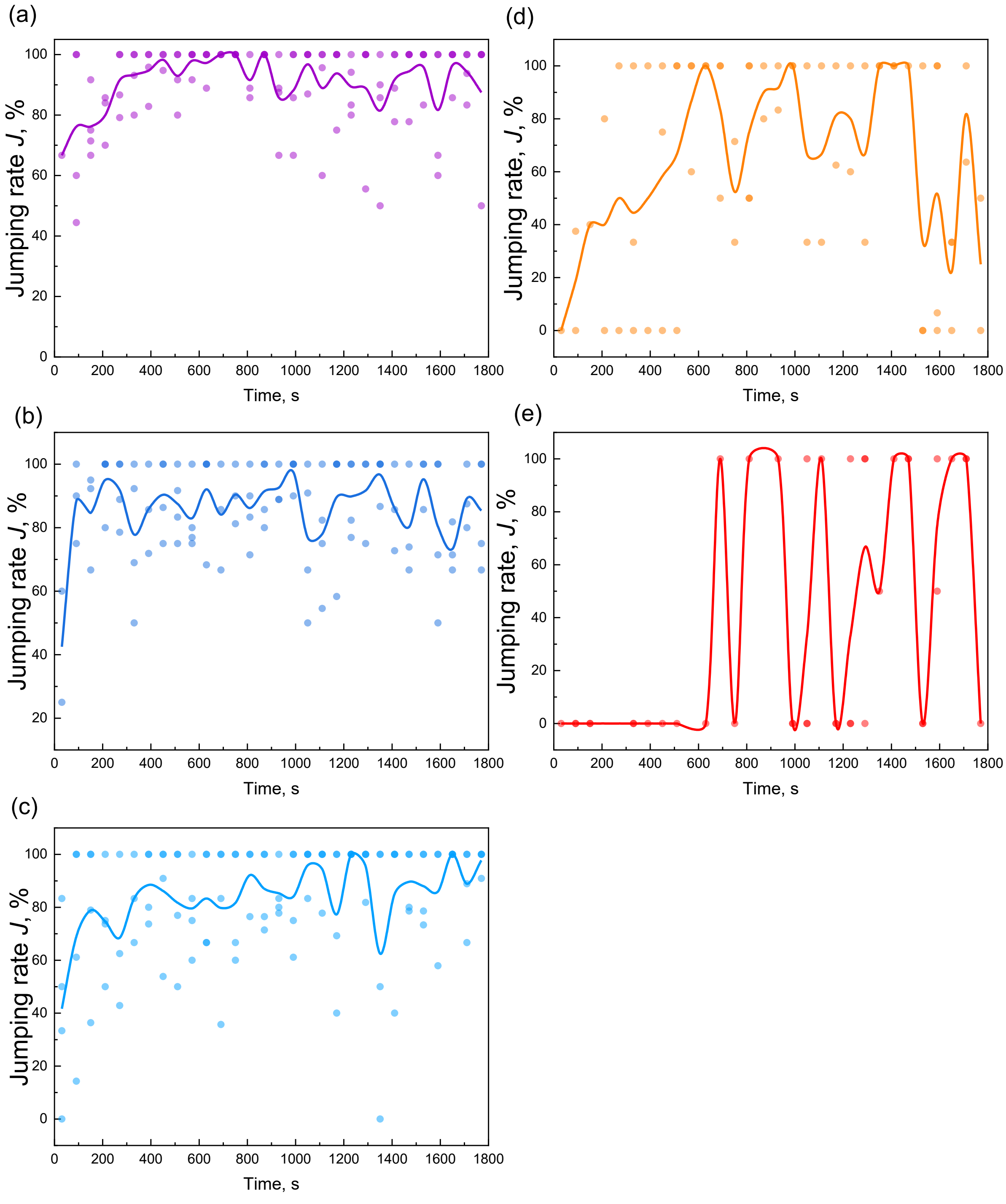

Figure S6. **Jumping rate (the proportion of jumping events relative to all coalescence events) as a function of time *t* (s)** on array of cones with variable spacing ("black silicon" and cones with 1, 1,5, 2, 3 μm spacing) from at least 4 independent measurements (*n* = 4…5) on different samples at similar conditions: (***a***) "black silicon" and hexagonally packed cones with (***b***) 1, (***c***) 1.5, (***d***) 2, and (***e***) 3 μm spacing. Sample with 4 μm spacing did not exhibit jumping therefore it is not presented.

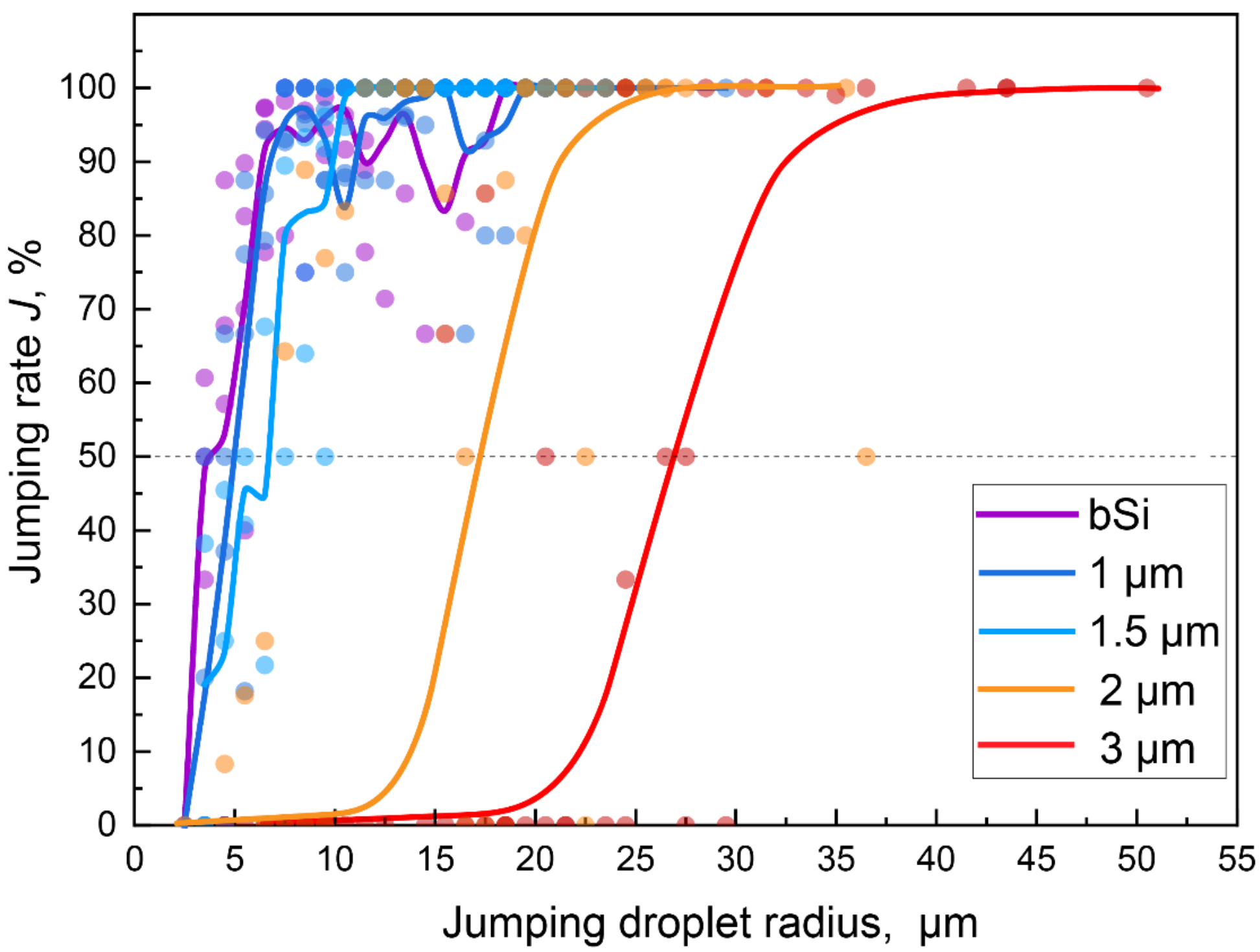

Figure S7. **Jumping rate (the proportion of jumping events relative to all coalescence events) as a function of the averaged radii of coalescing droplets (two or more).** on array of cones with variable spacing ("black silicon" and cones with 1, 1,5, 2, 3 µm spacing) from at least 4 independent measurements (n = 4…5) on different samples at similar conditions. Violet, blue and light blue curves are result of averaging of data, while orange and red curves are guide for an eye due to small number of jumps occurring on sparse cones. Dashed line at J = 50% was used to define critical jumping radius r_c.

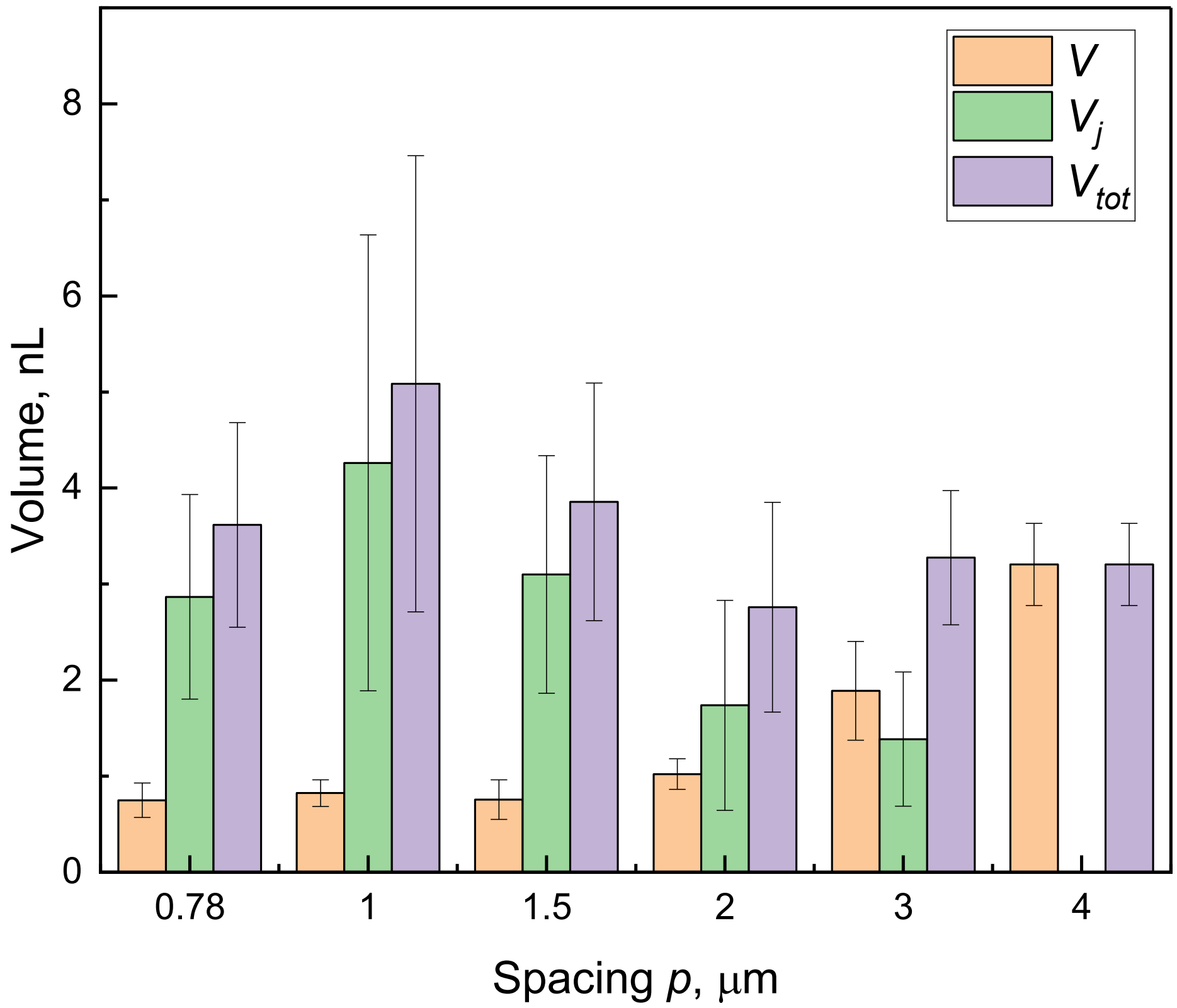

Figure S8. **Assessment of condensation performance in terms of volume** per sample area (0.154 mm^{-2}) on array of cones with variable spacing *p* ("black silicon" and cones with 1, 1,5, 2, 3 µm spacing): *V* volume of condensed water on the sample after 30 min of experiment (orange bar), V_j volume of removed water by jumping within 30 min of experiment, total amount of water condensed during the experiment – $V_{tot}=V+V_j$. Error bars correspond to the standard deviation of at least 4 independent measurements ($n = 4 \ldots 5$) on different samples at similar conditions.

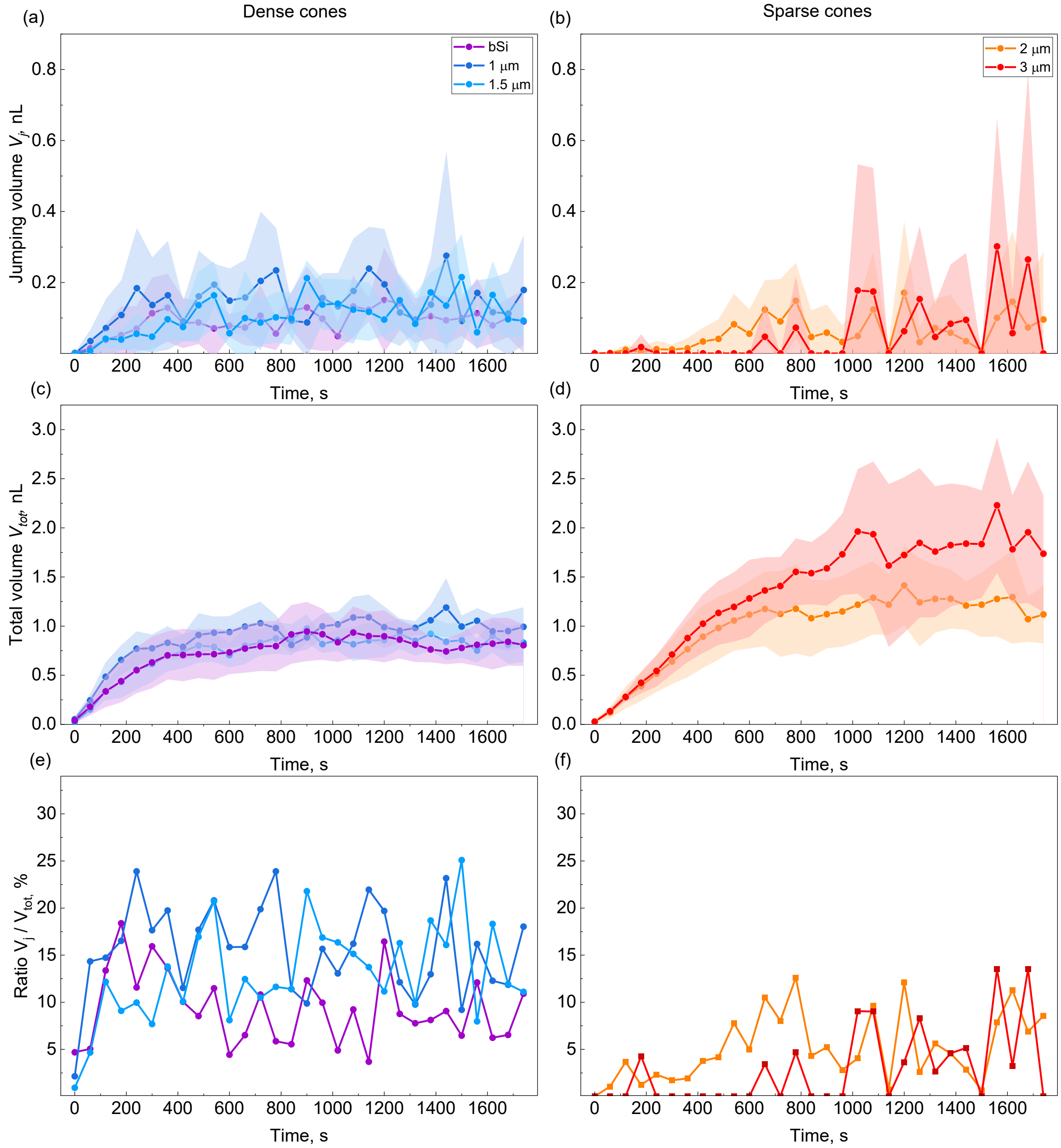

Figure S9. **Evolution of volume-related parameters** per sample area (0.154 mm^{-2}) for arrays of dense (black silicon and cones with 1 and 1.5 µm spacing; a, c, e) and sparse (2 and 3 µm spacing; b, d, f) cones. Shown are the jumping volume V_j(a, b), total volume $V_{\text{tot}} = V + V_j$(c, d), and their ratio V_j / V_{tot}(e, f). All graphs share the same time axis and legend. Error bars (shown as shaded regions) on a, b, c, d represent the standard deviation from at least four independent measurements (n = 4…5) performed on different samples under similar conditions.

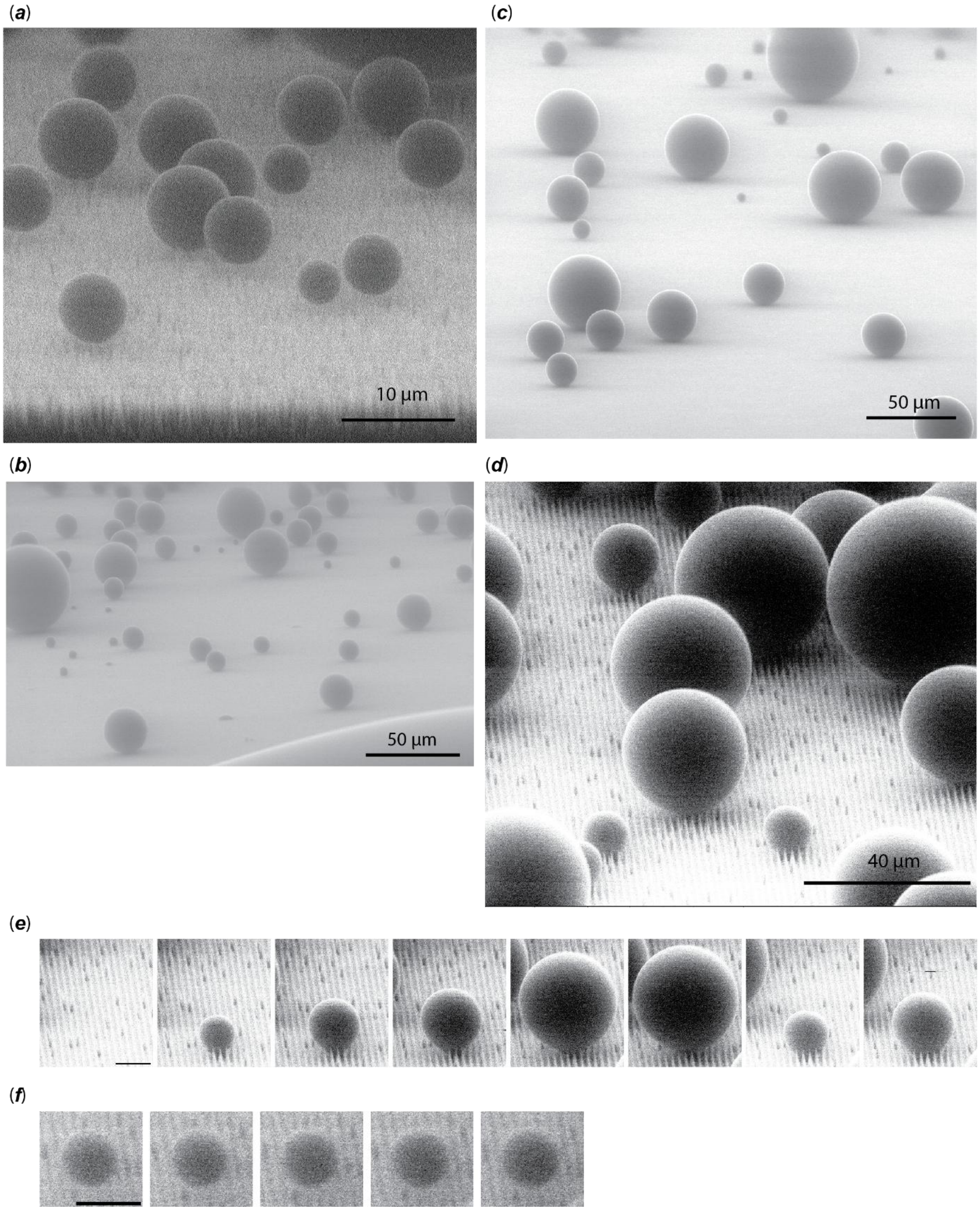

Figure S10. **ESEM micrographs of microdroplets on microstructured surfaces with variable spacing:** (***a***) "black silicon" and hexagonally packed cones with (***b***) 1, (***c***) 1.5, (***d***) 2 µm spacing. Growth of (**e**) partial Cassie droplets on sparse cones followed by droplet departure and a new growth cycle and (**f**) Cassie droplets. Scale bar is 10 µm.

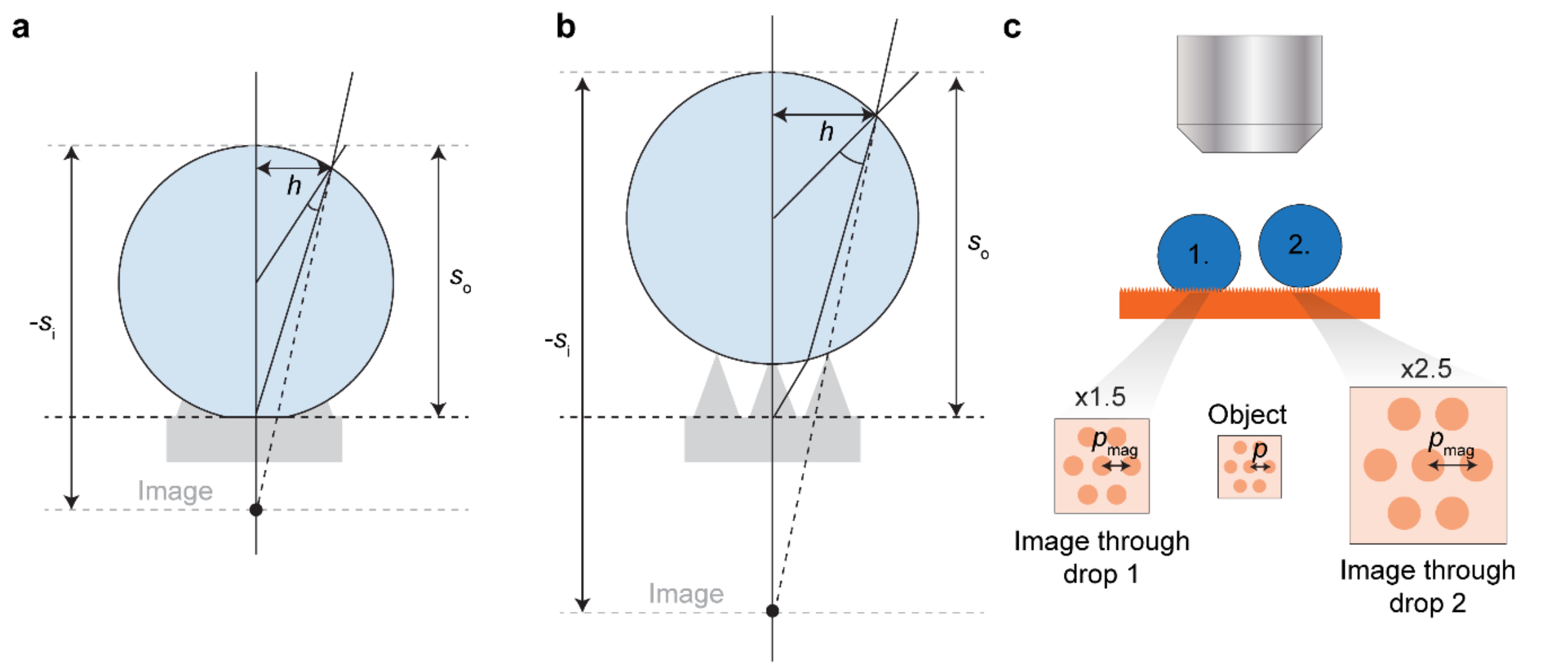

Figure S11. Magnification effect of microscopic droplets. (***a***) Optical geometry for Wenzel droplet, where light refraction at the top interface creates an image below the droplet. The image distance (s_i) is larger than object distance (s_o), resulting in positive magnification. (***b***) Optical geometry for Cassie droplets, where light refraction occurs at both top and bottom interfaces. This configuration increases the image distance resulting in greatest magnification effect for Cassie droplets. (***c***) Measuring the difference between microcone pitch p on dry surface and when imaged through droplet (p_mag) is used to quantify the magnification effect, which indicates the wetting state of the droplet.

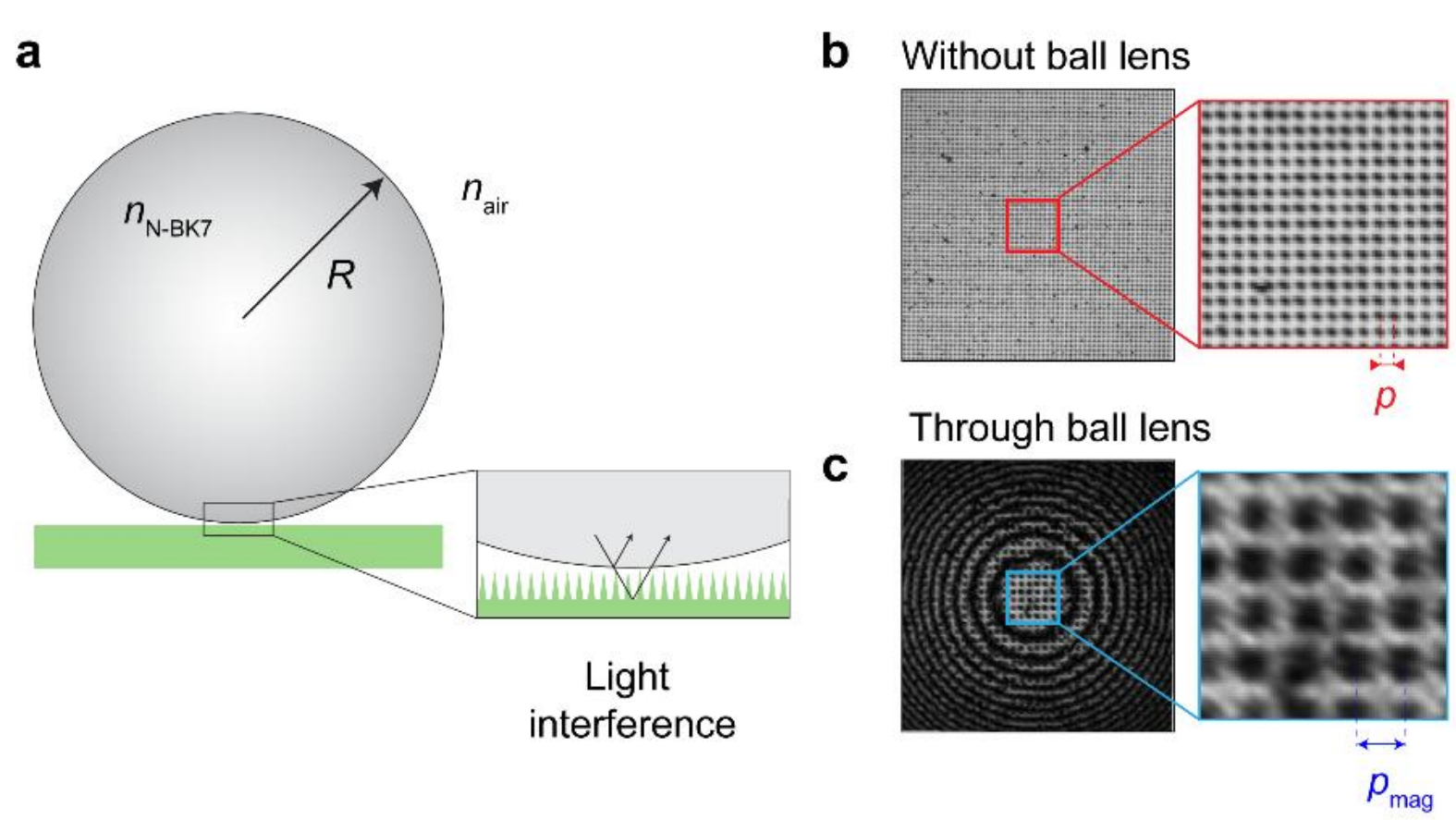

Figure S12. **Verification of the lens effect of condensing microscope using solid ball lenses. (*a*)** A scheme of a N-BK7 ball lens on a microcone sample (p = 2 µm). The inset shows light interference produced at the thin air layer between the microcone sample and curved ball lens. (***b***) A microscopy image of the microcone surface without the ball lens, and a zoomed region of the centre area. The distance p is 2 µm as expected. (***c***) A microscope image, and a zoomed region of the centre area, of the same sample as in (*b*) imaged through the ball lens, showing a magnified microcone sample with p_mag of 6.5 µm. The magnification is thus $M = p_\mathrm{mag}/p$ = 3.3x. The interference fringes shown in inset (***c***) are visible in this image.

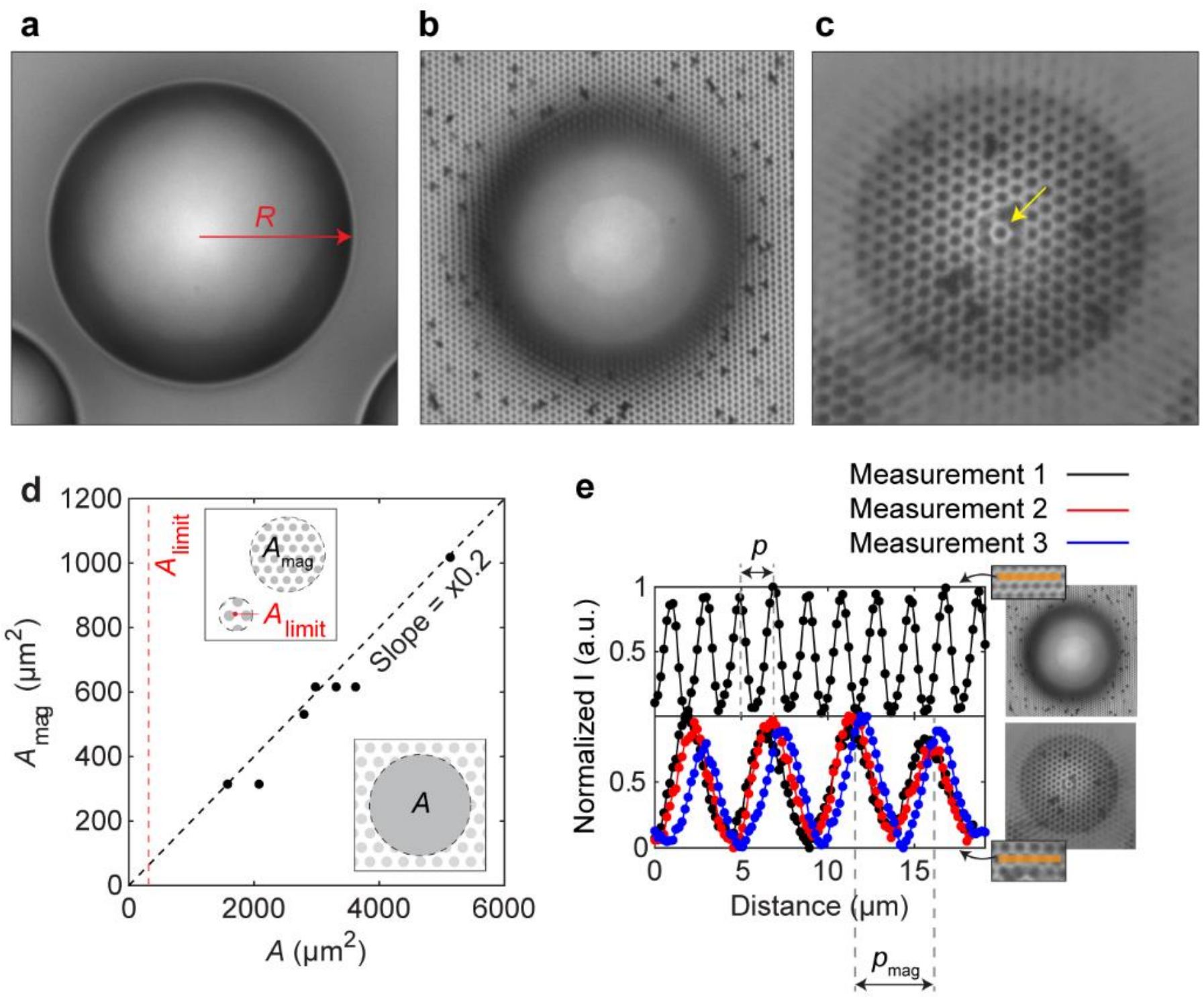

Figure S13. **Analysis of magnification factor form experimental data. (*a-c*)** Experimental microscopy images of the three z-planes used to analyze the magnification factor. The red arrow indicates drop R, and yellow arrow shows a contract region at the center of the droplet. (***d***) Experimentally determined visible through-droplet area A_{mag} as a function of droplet area A. A_{limit} show the smaller droplet area to resolve an area form which p_{mag} can be measured. (***e***) Examples of intensity as a function of distance plots used to measure p and p_{mag}.

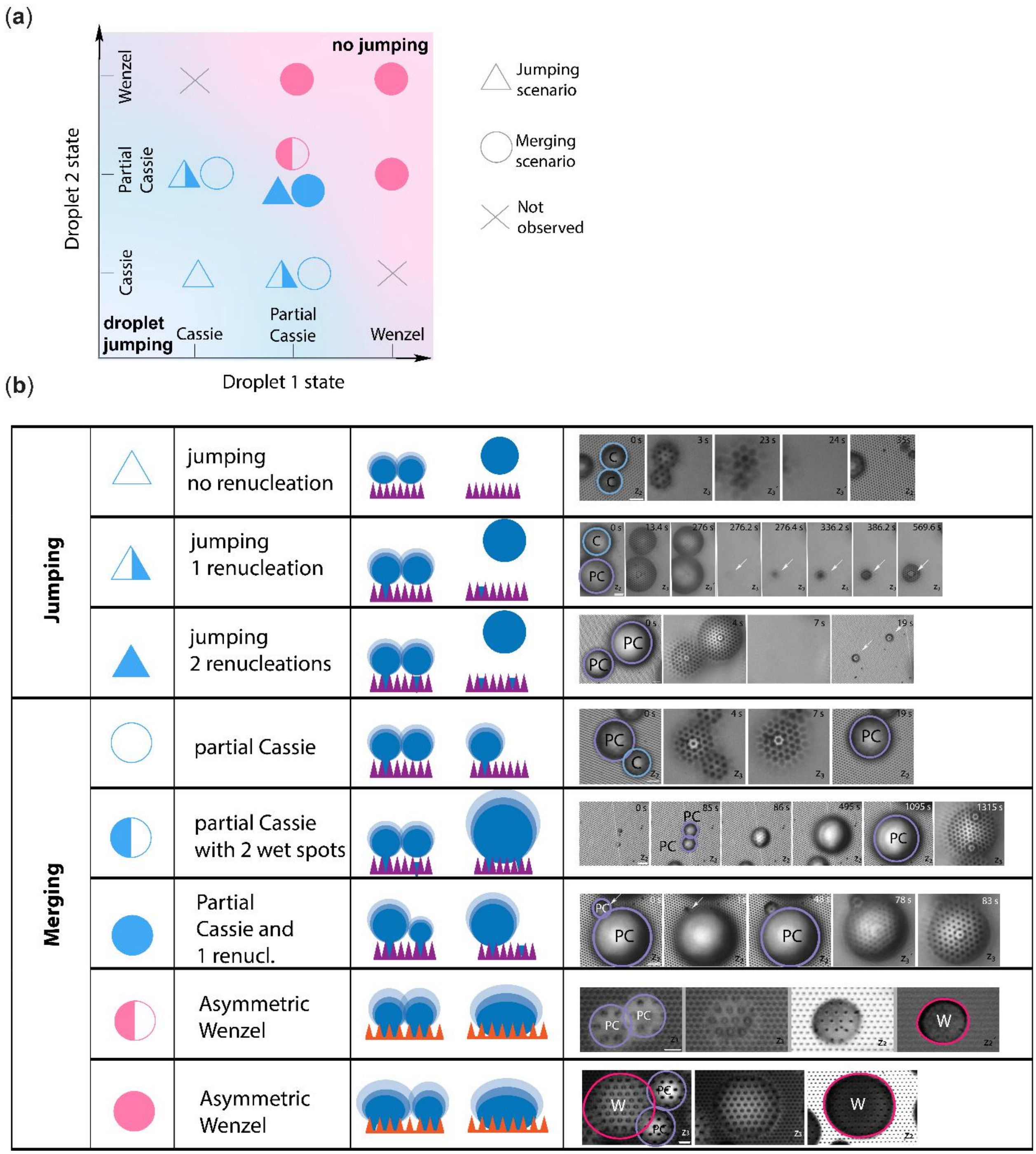

Figure S14. **Coalescence scenarios of droplets with different morphologies.** (***a***) Diagram illustrating the interaction between two droplets with distinct morphologies in the Cassie, partial Cassie, and Wenzel states resulting in various scenarios after coalescence. (***b***) Table illustrating various jumping and merging scenarios, including: the symbol used in the diagram, the resulting state after coalescence, a schematic of the coalescence process, and representative optical micrographs with the focal plane indicated. Scale bar is 10 µm. Blue circle labeled 'C' indicates Cassie droplets; violet circle labeled 'PC' indicates partial Cassie droplets; pink circle labeled 'W' indicates Wenzel droplets.

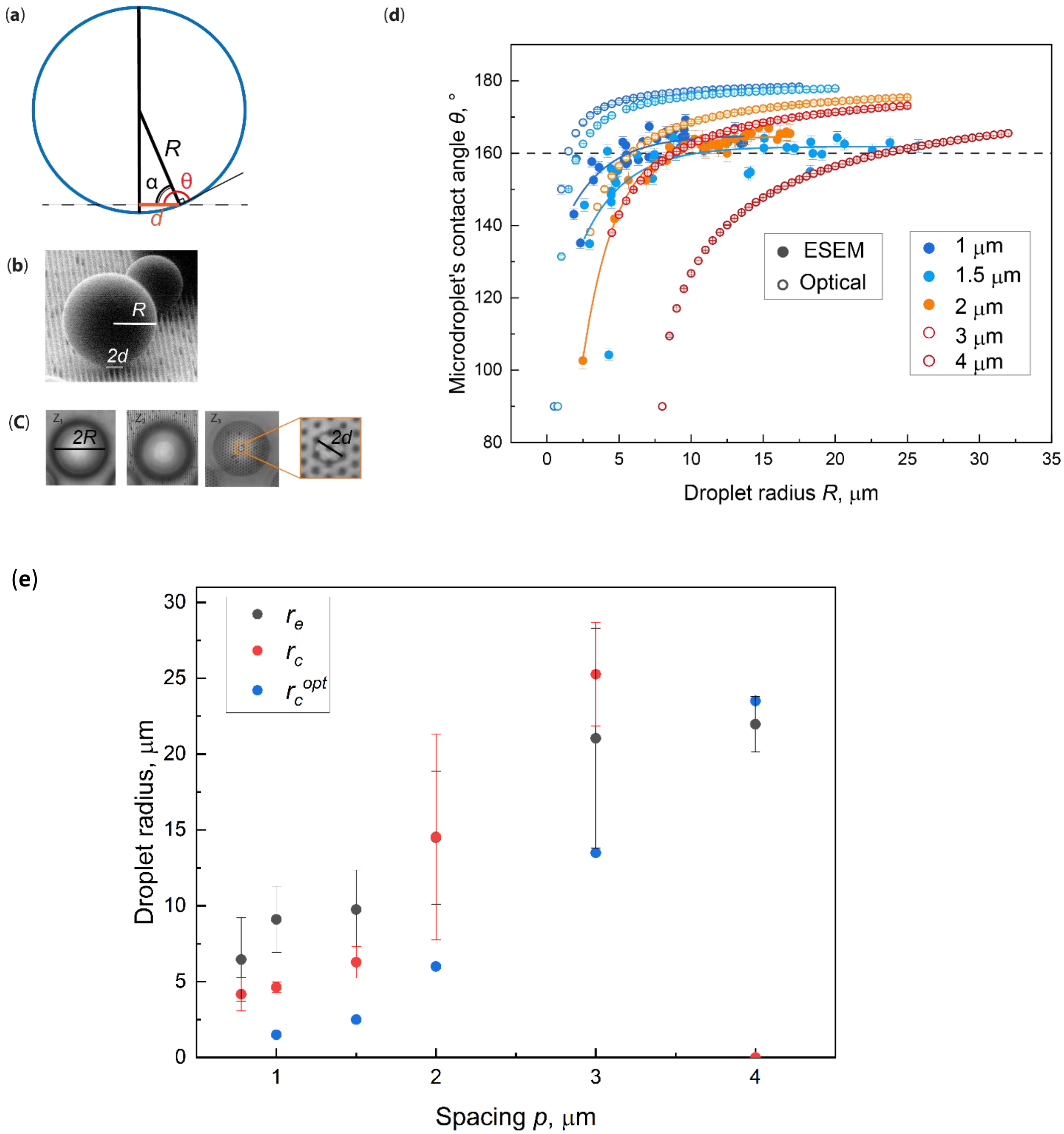

Figure S15. **Microdroplet's contact angle measurements and critical jumping radius**. (***a***) Schematics of contact angle measurement geometry; examples of contact angle measurement from (***b***) ESEM image, (***c***) Optical imaging through the droplet technique. (***d***) Comparison of microdroplet's contact angle measurements estimated from ESEM (filled circles), and prediction of contact angle based on size of wetted spot (hollow circles). (**e**) Predictions of critical jumping radius on array of cones with variable spacing p ("black silicon" and cones with 1, 1,5, 2, 3,4 µm spacing): experimental critical jumping radius r_c (red dots) vs. predicted coalescence radius r_e (black dots) and predicted jumping radius from optical microscopy with changing focus for partial Cassie droplets r_c^{opt} (blue dots). Error bars correspond to the standard deviation of at least 4 independent measurements ($n = 4\ldots5$) on different samples at similar conditions.

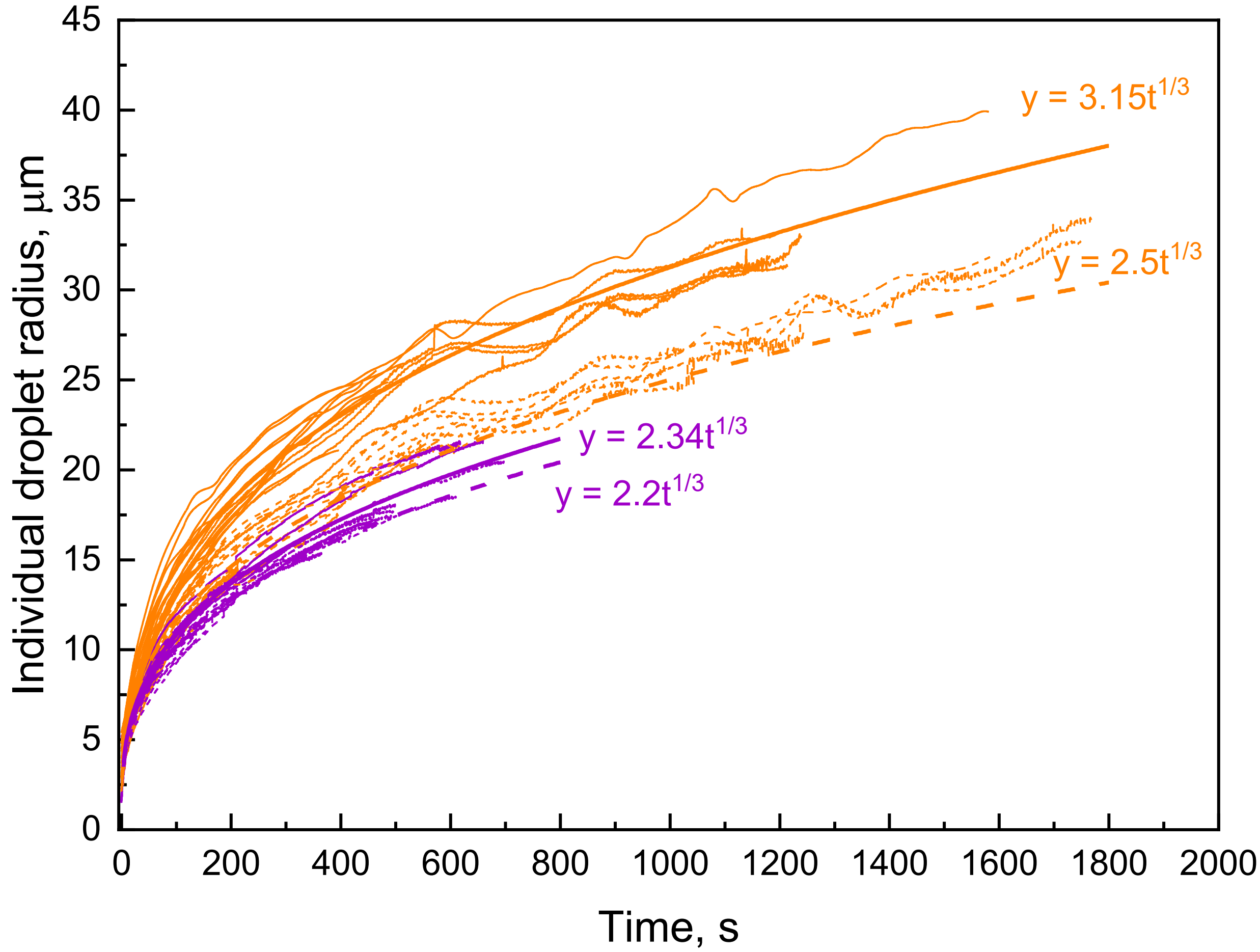

Figure S16. **Individual growth over time of partial Cassie and Cassie droplets.** Partial Cassie droplets on sparse cones with p = 2 µm (n = 24) are shown with orange lines fitted with $R(t) = 3.15t^{1/3}$ (thick line), while on "black silicon" are shown with violet lines (n = 9), fitted with $R(t) = 2.34t^{1/3}$(thick line). Cassie droplets on sparse cones are shown with dashed orange lines (n = 12), fitted with $R(t) = 2.5t^{1/3}$(thick dashed line), while Cassie droplets on "black silicon" are shown with dashed violet lines (n = 10), fitted with $R(t) = 2.2t^{1/3}$(thick dashed line).

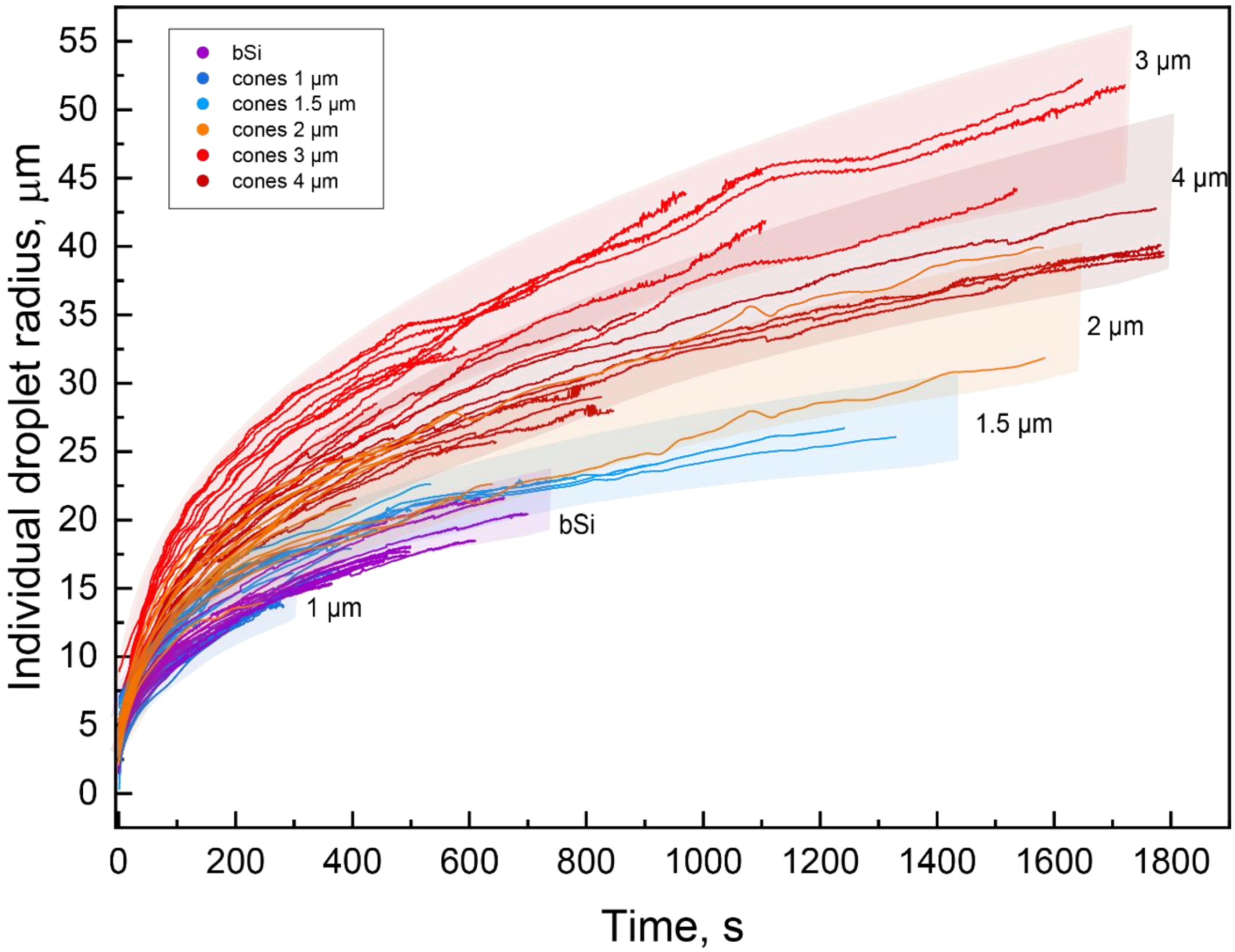

Figure S17. **Individual droplets growth** over time for "black silicon" and cones with 1, 1,5, 2, 3 and 4 μm spacing of at least 10 independent measurements (*n* = 10…20) for each sample type.

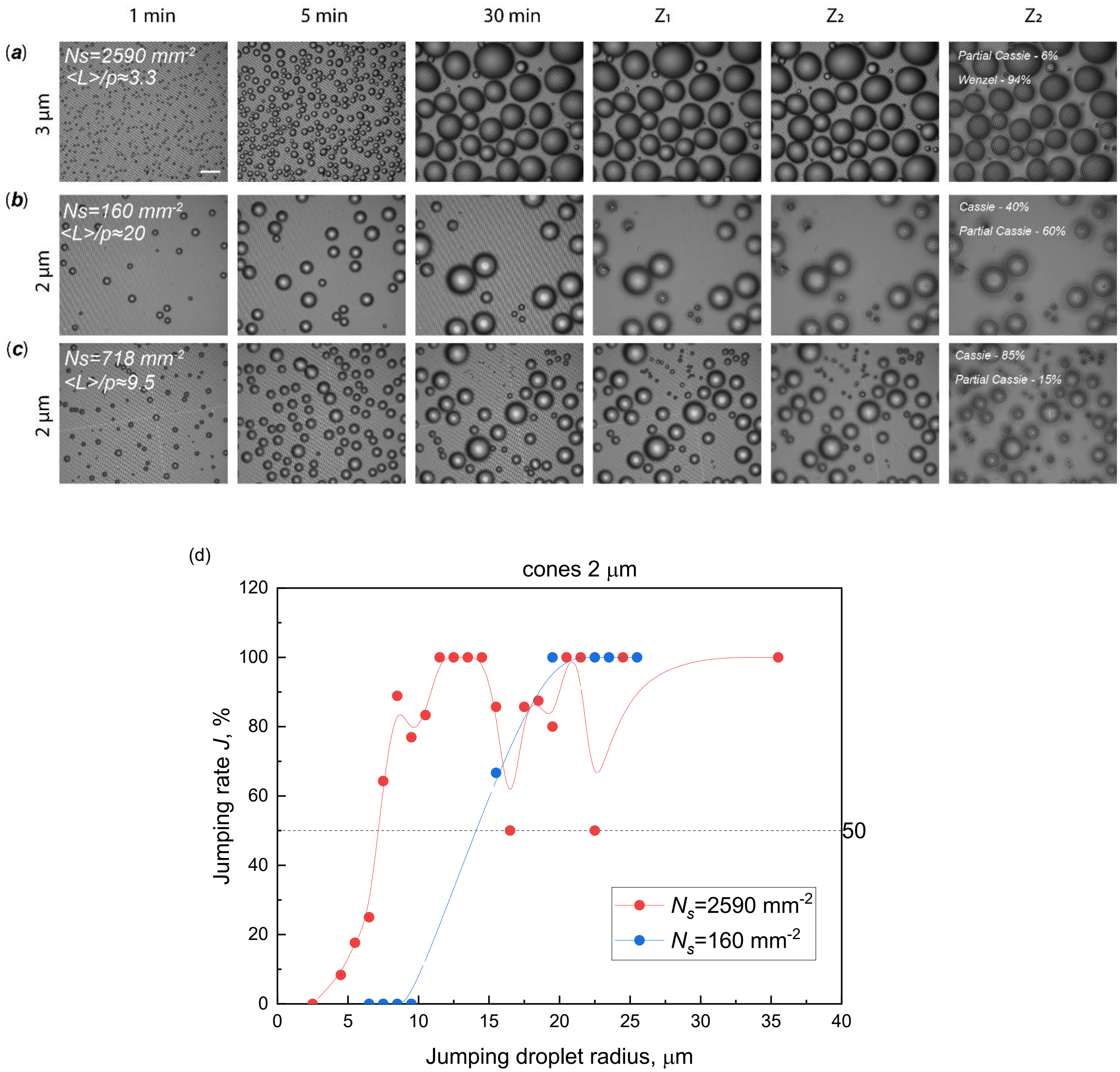

Figure S18. **Effect of nanoscale contamination on nucleation density N_s, jumping dynamics and droplet's morphology**. Breath figures after 1, 5, 30 min of condensation and variable focus optical imaging on microconical samples coated with PFOTS using protocol[3]. (***a***) Samples with 3 µm spacing, nucleation density N_s = 2590 mm^{-2} and $<L>/p \approx 3.3$ in Wenzel state; Samples with 2 µm spacing (***b***) with $N_s \approx$ 160 mm^{-2} and $<L>/p \approx 20$ exhibited inhibited jumping activity ($N_j \approx$ 97 mm^{-2}, $r_c \approx$ 15 µm), Cassie / partial Cassie ratio 40%/60% respectively (***c***) $N_s \approx$ 718 mm^{-2} and $<L>/p \approx 10$ showed enhanced jumping ($N_j \approx$616 mm^{-2}, $r_c \approx$ 6 µm). Cassie / partial Cassie ratio 85%/15% respectively Scale bar for ***a-c*** is 50 µm. (***d***) Jumping rate (the proportion of jumping events relative to all coalescence events) vs. jumping radius on 2 µm sample with nucleation density $N_s \approx$ 718 mm^{-2} (red) and 160 mm^{-2} (blue)

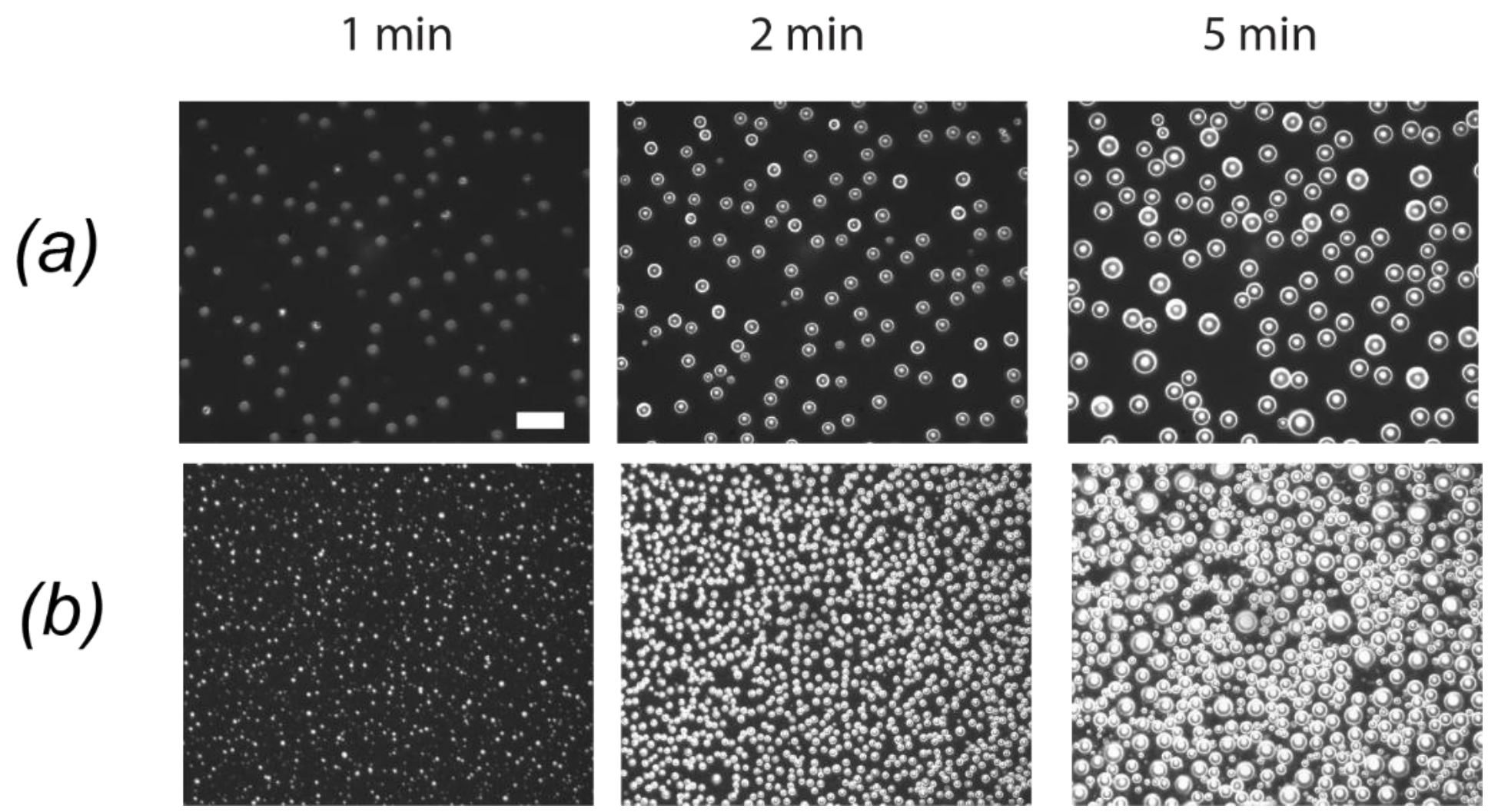

Figure S19. **Effect of deposition condition on nucleation density.** Breath figures after 1, 2, 5 min of condensation of bSi samples with PFOTS coating deposited with protocol [3] at ambient $RH = 31\%$ (***a***) and $RH = 58\%$ (***b***) having similar contact angles on planar surfaces: $\theta_A = 117.05 \pm 0.35°$, $\theta_R = 108.55 \pm 0.35$, CAH=8.5 ±0.3° and $\theta_A = 115.35 \pm 0.25°$, $\theta_R = 107.6 \pm 0.1°$, $CAH = 7.65 \pm 0.15°$ respectively. Scale bar is 50 μm.

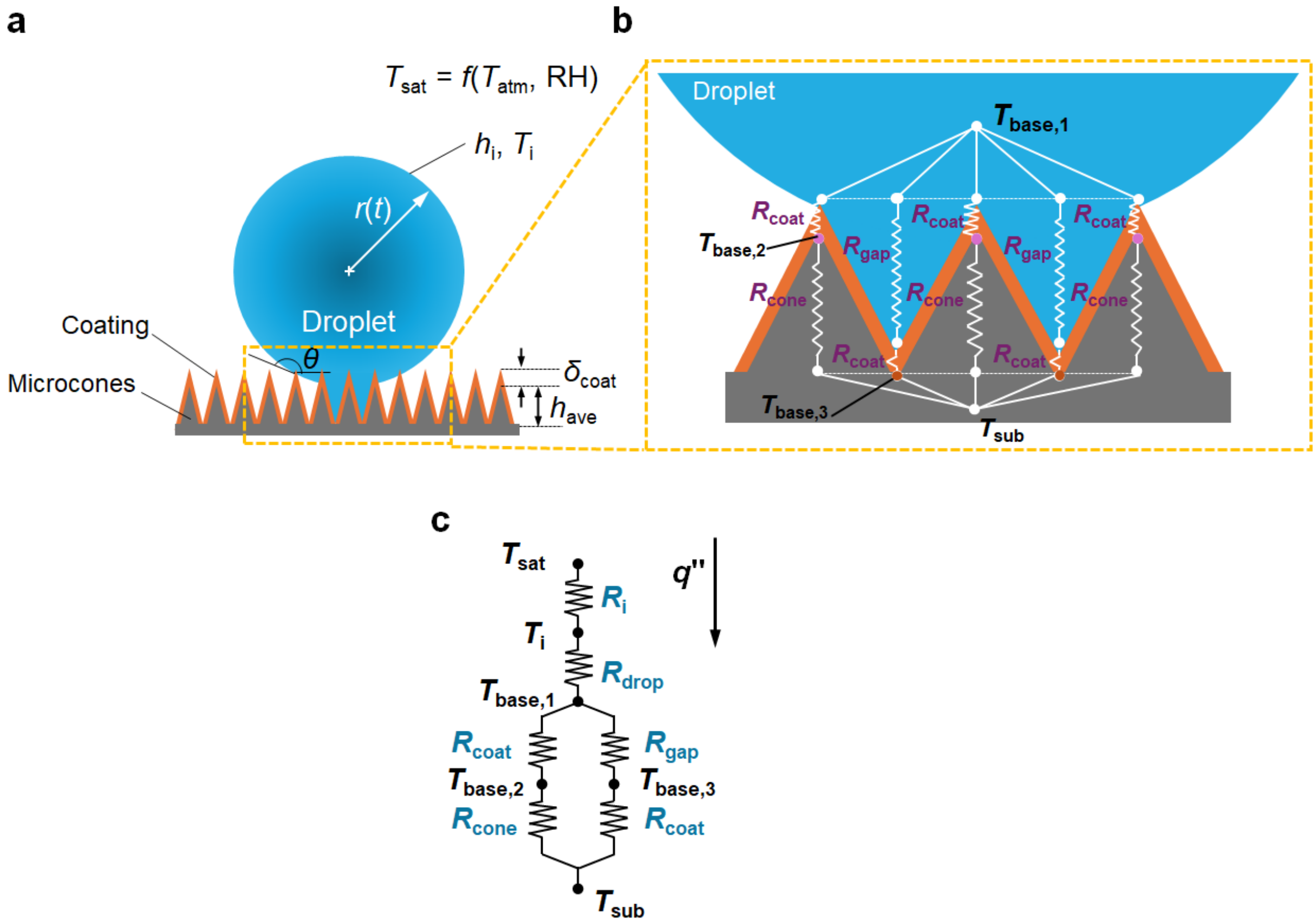

Figure S20. **Heat transfer analysis through a single droplet**. (***a***) Schematic of a droplet growing on the sample surface in partial Cassie state. (***b***) Thermal resistance network within the droplet and surface structure. The schematic shows the parallel path of heat flowing through the (i) coating and structure and (ii) droplet gap and coating. (***c***) Droplet thermal resistance diagram showing the droplet-vapor interface (R_i), droplet conduction (R_{drop}), coating (R_{coat}), surface structure (R_{cone}), and gap (R_{gap}) thermal resistances.

References

[1] S. Lepikko, V. Turkki, T. Koskinen, R. Raju, V. Jokinen, M. S. Kiseleva, S. Rantataro, J. V. I. Timonen, M. Backholm, I. Tittonen, R. H. A. Ras, *Small* **2024**, 2405335.
[2] M. Backholm, D. Molpeceres, M. Vuckovac, H. Nurmi, M. J. Hokkanen, V. Jokinen, J. V. I. Timonen, R. H. A. Ras, *Commun. Mater.* **2020**, *1*, 64.
[3] P. Koochak, M. S. Kiseleva, S. Lepikko, M. Latikka, R. H. Ras, W. S. Wong, *Adv. Mater. Interfaces* **2024**, 2400619.
[4] T. Huhtamäki, X. Tian, J. T. Korhonen, R. H. A. Ras, *Nat. Protoc.* **2018**, *13*, 1521.
[5] M. J. Hokkanen, M. Backholm, M. Vuckovac, Q. Zhou, R. H. A. Ras, *Adv. Mater.* **2021**, *33*, 2105130.
[6] V. Liimatainen, M. Vuckovac, V. Jokinen, V. Sariola, M. J. Hokkanen, Q. Zhou, R. H. A. Ras, *Nat. Commun.* **2017**, *8*, 1798.
[7] M. D. Mulroe, B. R. Srijanto, S. F. Ahmadi, C. P. Collier, J. B. Boreyko, *ACS Nano* **2017**, *11*, 8499.
[8] G. Wu, M. Hong, *Engineering* **2024**, *36*, 102.
[9] L. Chen, Y. Zhou, R. Zhou, M. Hong, *iScience* **2020**, *23*, 101211.
[10] E. Hecht, *Optics*, Pearson Education, Inc, Boston, **2017**.
[11] T. Mouterde, G. Lehoucq, S. Xavier, A. Checco, C. T. Black, A. Rahman, T. Midavaine, C. Clanet, D. Quéré, *Nat. Mater.* **2017**, *16*, 658.
[12] C. Ma, L. Chen, L. Wang, W. Tong, C. Chu, Z. Yuan, C. Lv, Q. Zheng, *Nat. Commun.* **2022**, *13*, 5381.
[13] N. Miljkovic, R. Enright, E. N. Wang, *J. Heat Transf.* **2013**, *135*, 111004.
[14] J. Liu, P. Faghihi, W. Yang, S. Yang, V. Ganesan, O. Gandhi, J. Zhang, W. Yang, N. Miljkovic, *Droplet* **2026**, *5*, e70056.
[15] P. Lecointre, S. Laney, M. Michalska, T. Li, A. Tanguy, I. Papakonstantinou, D. Quéré, *Nat. Commun.* **2021**, *12*, 3458.
[16] J. W. Rose, L. R. Glicksman, *Int. J. Heat Mass Transf.* **1973**, *16*, 411.
[17] C. Dietz, K. Rykaczewski, A. G. Fedorov, Y. Joshi, *Appl. Phys. Lett.* **2010**, *97*, 033104.
